# A review on radiochromic film dosimetry in radiation therapy

**DOI:** 10.1002/acm2.70365

**Published:** 2025-12-02

**Authors:** Arash Darafsheh, Hamid Ghaznavi

**Affiliations:** ^1^ Department of Radiation Oncology WashU Medicine St. Louis Missouri USA

**Keywords:** calibration, dosimetry, monomer, optical density, polymerization, radiochromic film

## Abstract

Radiochromic films (RCFs) are ubiquitous in radiation oncology clinical and research settings to measure the radiation dose over a two‐dimensional (2D) area due to their high spatial resolution, water‐equivalency, and relative ease‐of‐use. Upon irradiation, the constituent monomers of the active layer of RCFs undergo polymerization, leading to a visible darkening that enables quantitative dosimetry. Quantitative RCF dosimetry requires film calibration against a reference dosimeter. Film darkening depends on various radiation‐field and environmental parameters that need to be carefully considered to ensure the highest accuracy in film dosimetry. Several commercially available RCF models offer distinct dynamic dose ranges, allowing users to select appropriate options based on specific dosimetric needs. Here, we provide a review on the progress and practice of RCF dosimetry.

## 1 INTRODUCTION

Accurate measurement of radiation dose is a critical component of quality assurance (QA) in radiation therapy. Various dosimeters, such as ionization chambers,^1^ semiconductor dosimeters,^2^ thermoluminescent dosimeters (TLDs),^3^ optically stimulated luminescent dosimeters (OSLDs),^4^ scintillators,^5^, ^6^ and radiochromic films (RCFs),^7^, ^8^, ^9^, ^10^, ^11^, ^12^, ^13^, ^14^ support different dosimetry needs in radiation therapy. Each of these technologies has its own advantages and limitations.^15^ RCFs are ubiquitous in radiation oncology clinical and research settings to measure the radiation dose in simple and complex fields with high spatial resolution over a two‐dimensional (2D) area. Although devices based on arrays of ionization chambers and diodes or scintillation imaging techniques can provide 2D dosimetry, the high spatial resolution, relative ease‐of‐use, and water‐equivalency of RCFs make them appealing for 2D and small field dosimetry applications. Their use in dosimetry and QA spans megavoltage x‐ray,^9^, ^10^, ^11^ electron,^16^, ^17^ proton,^18^, ^19^ and heavy ion^20^, ^21^ therapies, as well as magnetic resonance imaging‐guided radiation therapy (MRIgRT),^22^, ^23^ brachytherapy,^24^, ^25^ radionuclides,^26^, ^27^ and kilovoltage x‐ray irradiators.^8^ Unlike radiographic films,^28^ RCFs exhibit minimal energy dependence due to their water‐equivalent characteristics, and they do not need wet chemical processing. RCFs are used in many aspects of radiation therapy dosimetry, such as radiation leakage check and commissioning of clinical accelerators, routine QA of radiation therapy machines, patient‐specific QA, small‐field dosimetry, and in vivo dosimetry. Research applications of RCFs include dose verification for small animal irradiators and in vitro studies. RCFs have proven useful for dosimetry of developing radiation therapy modalities, such as ultra‐high dose rate (UHDR) or FLASH radiotherapy,^29^, ^30^, ^31^ very‐high energy electron,^32^ and spatially fractionated radiation therapies.^33^, ^34^, ^35^

Several commercially available RCF models offer distinct measurement dose ranges, allowing users to select a suitable option based on specific dosimetric needs. The principle of operation of RCFs is based on radiation‐induced polymerization of the active layer of the film.^10^, ^12^ The active layer of the films is composed of diacetylene monomers sandwiched between two protective layers or coated over a base layer, depending on the film design. Initiations, polymerization, and termination are three steps that occur during the response of the film to radiation. Exposure to ionizing radiation results in covalent crosslinking of polymer chains, where the level of crosslinking increases proportionally with the dose received. Polymerization leads to visible film darkening. In order to perform quantitative film dosimetry, the change in darkening is quantified by measuring the transmission or absorption of the films through the optical density (OD), which represents the logarithmic ratio of the incident to transmitted light intensity, to establish a calibration curve.^36^ RCF darkening depends on various radiation field and environmental parameters that need to be carefully considered to assure highest accuracy in film dosimetry.

In this paper, we provide a review on the progress and practice of RCF dosimetry offering a comprehensive and structured summary of the developments in RCF dosimetry, from film selection and calibration to practical considerations in advanced clinical applications. The structure of the paper is as follows. In Section 2, a brief historical overview of RCF development is provided. Physical and chemical properties of RCFs are discussed in Section 3. Section 4 covers the considerations in quantitative RCF dosimetry including film handling and preparation, calibration, and readout systems such as 2D scanners and spectrophotometers. Factors affecting the film response, such as dose, dose rate (conventional and ultra‐high), energy, linear energy transfer (LET), manufactured batch, post‐irradiation time, film orientation, lateral response artifact (LRA), temperature and humidity, mechanical stress and light, and external magnetic fields, are discussed in detail in Section 5. Uncertainty analysis in RCF dosimetry is provided in Section 6. Section 7 is the Conclusion of the paper.

## 2 HISTORY AND DEVELOPMENT OF RADIOCHROMIC FILMS

RCFs belong to a class of polymer‐based dosimeters that change color upon irradiation.^7^, ^8^, ^9^, ^10^, ^11^, ^12^ A review on historical development of RCFs has been provided by Butson and Niroomand‐Rad.^37^ It is interesting to note that the history of “radiochromic materials” precedes the discovery of x‐rays. In the early 19^th^ century, Niepce demonstrated a radiochromic process (crosslinking in an unsaturated hydrocarbon polymeric mixture upon light exposure) in a light‐sensitive material (“bitumen of Judea”) for photography or “heliography” as he named it.^38^ This process required 8–10 hours of continuous sunshine exposure which motivated further research into radiochromic materials capable of more direct imaging. By the middle of the 19^th^ century, a more direct imaging process based on photo‐reduction in potassium dichromate fused in gels and papers had been developed. Since then, photographic imaging using organic systems undergoing photo‐polymerization process was developed further, a process in which irradiation generates free radicals that initiate the formation of covalently bonded, crosslinked carbon chains.^39^ Another class of organic imaging materials combines this photopolymerization mechanism with leuco dyes, which undergo a color change in response to radiation exposure.

The use of radiochromic materials in the context of ionizing radiation imaging and dosimetry dates to the early 20^th^ century when pastille discs made of barium platinocyanide were used to estimate the radiation absorbed dose defined as certain amount of radiation that can cause skin erythema.^37^ By the middle of the 20^th^ century, radiation imaging using systems incorporating triphenyl tetrazolium chloride salts^40^, ^41^, ^42^, ^43^, ^44^ and triphenylmethane leucocyanides salts^45^ were demonstrated. During the latter half of the 20^th^ century, solid solutions of triphenyl methane derivatives (crystalline polyacetylenes, particularly diacetylene) were developed as radiochromic media.^46^, ^47^ The National Institute of Standards and Technology (NIST) in the United States is responsible for most of the early research and development of radiochromic dosimetry materials during 1960's to 1980's mainly for industrial applications, such as food irradiation and sterilization dealing with kGy to MGy doses.

Since the mid‐1980s, International Specialty Products (ISP, Wayne, NJ, USA), a division of GAF (General Aniline & Film) Chemical Corporation, is responsible for most of the commercial developments of RCFs. During 1990's McLaughlin et al. reported on the use of RCFs for radiation therapy dosimetry.^48^, ^49^, ^50^ Since then, ISP (acquired by Ashland Inc. in 2011) has manufactured various generations of radiochromic films, also called Gafchromic™ films, with different structures supporting various dynamic ranges.

To address issues such as limited sensitivity, poor uniformity, size constraints, and high production costs in earlier models, ISP introduced the EBT film in 2004. In 2009, the EBT2 model was introduced with a single layer in which a yellow dye had been added to the active components. In 2011, the EBT3 model was introduced with symmetric design and addition of silica microparticles to diminish the Newton ring artifacts associated with the EBT2 model. In 2015, the EBT‐XD model with smaller nanocrystals in the active layer (lower sensitivity) was launched to support stereotactic body radiation therapy (SBRT) and stereotactic radiosurgery (SRS) dosimetry needs. In 2021, the EBT4 model was introduced to improve the nonuniformities of the EBT3 model.

## 3 PHYSICAL AND CHEMICAL PROPERTIES OF RCFS

### 3.1 Structural components

As mentioned earlier, various Gafchromic™ film models with their associated dynamic ranges are in the market to support radiation therapy dosimetry needs (Table 1). According to the vendor, EBT3/EBT4,^51^, ^52^, ^53^, ^54^ EBT‐XD,^55^, ^56^, ^57^, ^58^, ^59^ MD‐V3,^60^, ^61^, ^62^, ^63^ and HD‐V2^62^, ^63^, ^64^ models have dynamic ranges of 0.1–10 Gy, 0.4–40 Gy, 1–100 Gy, and 10–1000 Gy, respectively, due to their different sizes of monomer crystals in the active layer which allows them to cover a certain range of doses. The active layer thickness varies among different film models, measuring approximately 28 µm in EBT3 and EBT4, 25 µm in EBT‐XD, 15 µm in MD‐V3, and 8 µm in HD‐V2 films.^10^ The overall film thickness is approximately 0.28 mm for EBT3, EBT4, EBT‐XD, and MD‐V3, whereas the HD‐V2 model has a thinner profile of approximately 0.11 mm.

**TABLE 1:** Characteristics of common RCFs for radiation therapy applications.

Model	Dose (Gy)	Design	AL (µm)	S1 (µm)	S2 (µm)	AL's Z_eff_	Overall Z_eff_
EBT3	0.2–10	Type 3	28	125	125	7.46	6.71
EBT‐XD	0.4–40	Type 3	25	125	125	7.42	6.70
MD‐V3	1–100	Type 2	15	125	125	7.63	6.68
HD‐V2	10–1000	Type 1	8	97	N/A	7.63	6.73

AL: Active layer; S1: Substrate #1; S2: Substrate #2; Z_eff_: effective atomic number.

**FIGURE 1:**
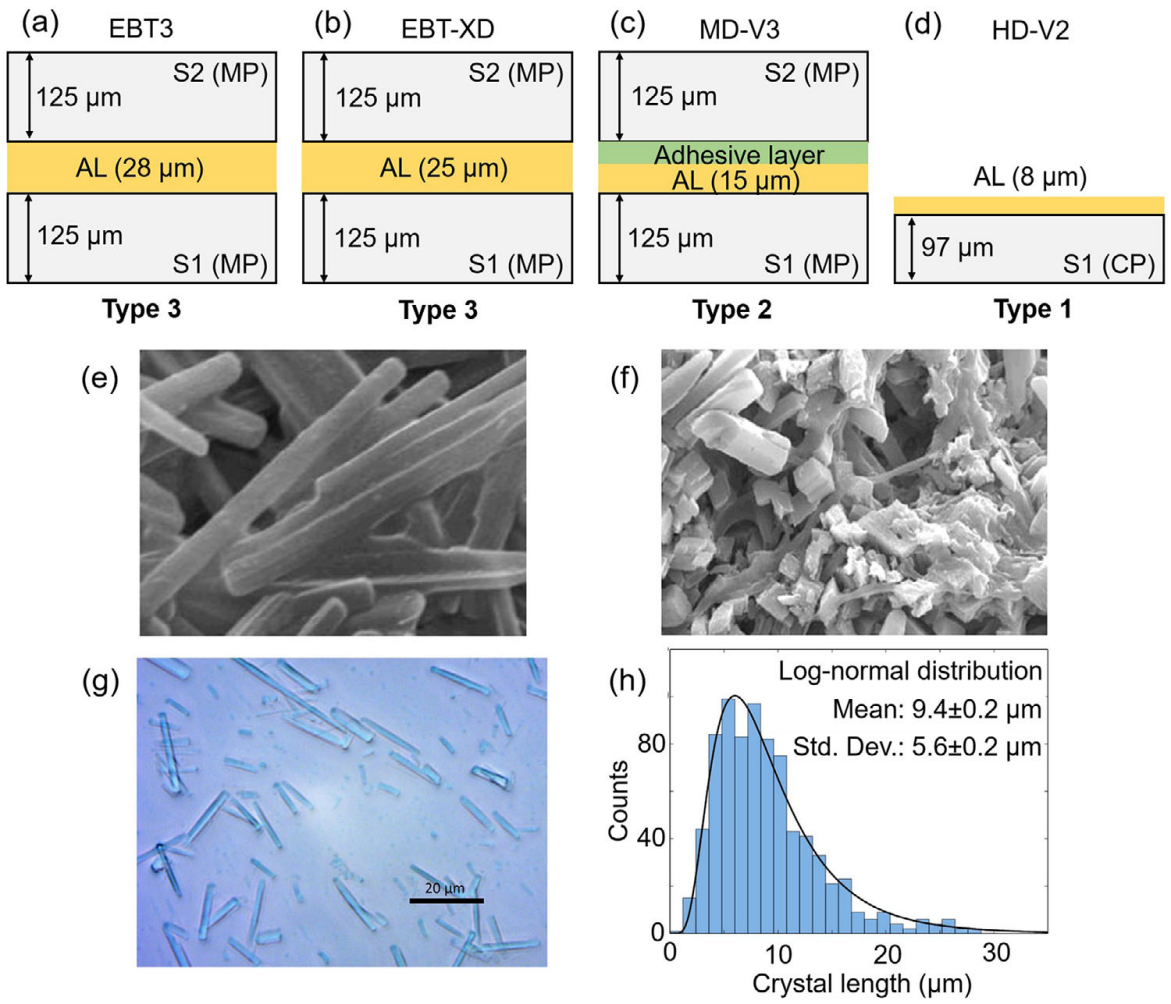
Structural schematics of (a) EBT3, (b) EBT‐XD, (c) MD‐V3, and (d) HD‐V2 RCFs. X‐ray crystallography of the active layer in (e) EBT2/EBT3 and (f) EBT‐XD models,^10^ along with (g) a microscopic image displaying monomer microcrystals in the EBT3 film's active layer and (h) their corresponding length distribution.^65^ The scale bar in panel (g) is 20 µm.

RCFs are constructed in multilayered formats, typically comprising one or more polyester substrates and a central radiation‐sensitive region known as the active layer (AL).^7^ There are three configuration types depending on the number of substrates and employment of an adhesive layer as shown in Figure 1a‐d. RCFs are commonly designed with a laminated architecture, where the active layer is enclosed between protective polyester sheets. The specific design of these laminations is influenced by manufacturing cost and technical constraints, which are proprietary to the manufacturer. HD‐V2 and unlaminated EBT3 films utilize a Type 1 configuration, making them well‐suited for radiation dosimetry that are highly susceptible to attenuation by polyester materials.^9^, ^37^ To provide mechanical protection and reduce the effects of ambient light exposure, both Type 2 and Type 3 film designs feature polyester substrates on both sides of the active layer. In Type 2 configurations, as seen in MD‐V3 and EBT2 films, the second polyester layer is attached using either a pressure‐sensitive acrylic adhesive or a water‐soluble polymer. The EBT3, EBT4, and EBT‐XD films utilize a Type 3 configuration, where the active layer is directly laminated between two polyester substrates without the use of any adhesive layers.^10^

#### 3.1.1 Substrate layer

Type 2 and Type 3 configurations of RCFs are constructed with two polyester substrates, Substrate #1 and Substrate #2, designed to support and protect the sensitive active layer. The composition of Substrate #1 depends on the specific application of the film. It may be made from smooth transparent polyester, such as in MD‐V3 and EBT2 models, or feature a surface treatment of microscopic silica particles (< 10 µm) as in the EBT3 and EBT‐XD models. These silica particles help to eliminate Newton's ring artifacts during scanning (a manifestation of the wave nature of light that happens due to an interference of multiple reflections of light between the film and the scanner's glass bed) by maintaining a small air gap between the film and the scanner's glass bed. In radiology‐oriented films such as RTQA2, XR‐RV3, and XR‐QA2, Substrate #1 is made from opaque white polyester infused with barium sulfate, which increases the photoelectric absorption of kilovoltage photons, thereby enhancing the film's sensitivity. These films are typically scanned in reflection mode. Substrate #2, the over‐laminate layer, may also be transparent and smooth (as in MD‐V3 and EBT2), silica‐coated (EBT3 family and EBT‐XD), or contain a yellow dye (RTQA2, XR‐RV3, XR‐QA2). This yellow dye filters out UV and blue light—wavelengths most likely to affect the sensitive active layer—and visually enhances the color change produced after radiation exposure. However, it also narrows the usable spectrum for blue and green channels, making triple‐channel dosimetry less effective in such films.^7^ Triple channel dosimetry is a form of multichannel analysis that uses all three scanner color channels (R,G,B) simultaneously to determine dose from a scanned RCF image^66^; instead of using one channel alone (e.g., R), the three channels are combined so the dose‐dependent signal can be separated from dose‐independent disturbances.

#### 3.1.2 Adhesive layer

In Type 2 RCF configurations, an adhesive layer is used to bind Substrate #2 to the active layer. This bonding is achieved using either a pressure‐sensitive acrylic adhesive or a water‐soluble polymer. Though not directly involved in the dosimetric function of the film, the adhesive layer plays a crucial structural role in maintaining the integrity and consistency of the multilayered film design, ensuring that the film components remain securely in place during handling and irradiation.^11^, ^37^

#### 3.1.3 Active layer

The active layer is the functional core of all RCFs. The dosimetric compound is embedded as microcrystalline structures within a matrix composed of a water‐soluble polymer. This layer is responsible for the film's response to ionizing radiation and is designed to provide reliable and reproducible dose measurements. To enhance energy independence across a wide photon energy, some film types, such as EBT2, EBT3/EBT4, HD‐V2, MD‐V3, and EBT‐XD, incorporate aluminum oxide nanoparticles within the active layer.^67^ These nanoparticles improve dosimetric consistency across different energy levels. A yellow marker dye is also often added; it remains stable even beyond 500 Gy and serves two critical purposes: compensating for minor thickness variations and enabling triple‐channel dosimetry when scanned using RGB flatbed scanners.^66^, ^67^, ^68^ In this context, the blue channel reflects film thickness and uniformity, while the green and red channels correlate more directly with absorbed radiation dose. Some specialized films use different materials in the active layer to achieve specific goals. For instance, the XR‐QA2 film includes bismuth oxide, which enhances its sensitivity to low‐energy kilovoltage photons, allowing precise dose measurements in the mGy range. However, this modification reduces its effectiveness at megavoltage energies compared to other RCFs.^7^

The dosimetric function of the active layer primarily arises from diacetylene compounds, particularly pentacosa‐10,12‐diynoic acid (PCDA) and its lithium‐substituted form (Li‐PCDA). These molecules polymerize upon exposure to radiation, forming an intensely colored product suitable for both visual inspection and quantitative analysis.^69^ Earlier models, like HD‐810 and MD‐55, used PCDA and covered high‐dose ranges (10–1000 Gy and 1–100 Gy, respectively). However, their limited sensitivity restricted their utility in lower‐dose applications. The introduction of Li‐PCDA improved sensitivity and enabled the development of modern RCFs like the EBT series,^7^ capable of measuring doses from a few cGy to 40 Gy or more. Furthermore, variations in the crystalline structure of Li‐PCDA influence the optical absorbance of the films. For example, EBT3 films have dominant absorption peaks at 633 and 595 nm, whereas HD‐V2 films absorb more strongly at 670 and 635 nm, influencing their dosimetric accuracy and energy response.^70^

EBT3 and EBT‐XD films are notable for their symmetrical construction, featuring a sensitive Li‐PCDA layer sandwiched between matte polyester layers. EBT3 includes silica particles in the protective layer to improve handling, while EBT‐XD features a thinner active layer, allowing for a broader dynamic range (up to 40 Gy vs. 10 Gy for EBT3). X‐ray crystallography of the active layer in EBT3/EBT2 and EBT‐XD films are shown in Figure 1e,f. Smaller crystal sizes in the EBT‐XD model leads to less sensitivity allowing coverage of a higher dynamic range. These attributes make EBT‐XD particularly suitable for high‐dose applications such as SRS and SBRT dosimetry. Both films are considered energy‐independent for megavoltage photon and electron beams but still require individual calibration due to inherent model‐to‐model and batch‐to‐batch variations.^54^, ^58^

### 3.2 Chemical composition of RCFs

**FIGURE 2:**
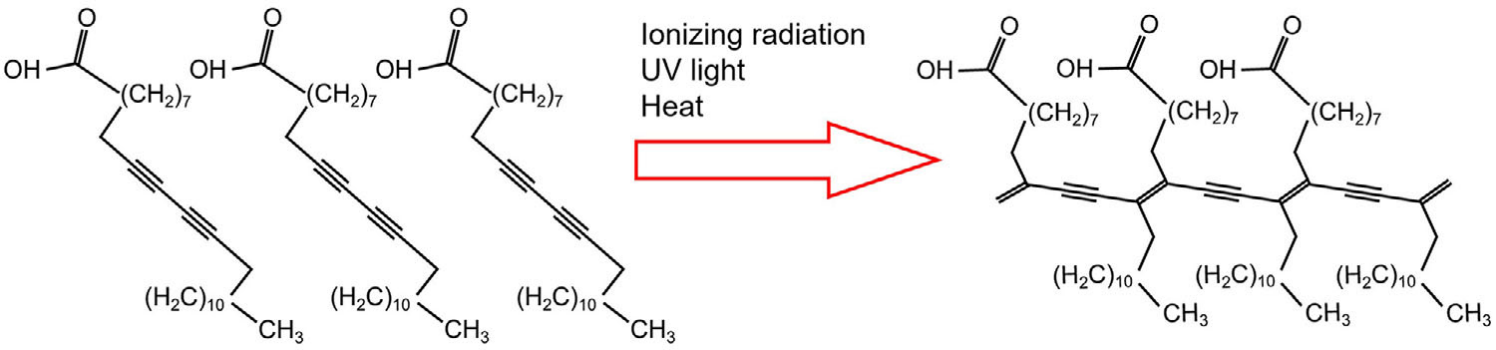
Polymerization reaction of pentacosa‐10,12‐diynoic acid induced by external stimuli such as ionizing radiation, UV light, or heat.^65^

The chemical properties of RCFs are primarily defined by the composition and behavior of their active layer. Central to this layer are diacetylene compounds, such as pentacosa‐10,12‐diynoic acid and its more sensitive derivative lithium pentacosa‐10,12‐diynoate.^37^ Upon exposure to ionizing radiation (as shown in Figure 2), these molecules undergo a solid‐state polymerization reaction, forming long‐chain conjugated polymers. Exposure to radiation induces energetic radicals that trigger 1,4‐trans addition polymerization of the embedded diacetylene monomers. This process results in a visible color change, a key chemical transformation that underpins RCF dosimetry.^71^, ^72^

Many modern RCFs also incorporate a yellow marker dye in their active layer that remains chemically stable under irradiation. The elemental composition of the sensitive layer plays a crucial role in film performance and is unique to each Gafchromic^TM^ model. Tables III and IV of the American Association of Physicists in Medicine (AAPM) Task Group (TG) 235^7^ summarizes the atomic percentages of key elements in the active layers of commonly used films for radiation therapy and diagnostic radiology. Notably, XR‐QA2 incorporates a significant amount of bismuth (Bi), resulting in a much higher effective atomic number (Z_eff_) compared to other models, which enhances its response to low‐energy x‐rays.

The solubility and uniform dispersion of these active compounds within the water‐soluble polymer matrix are critical for achieving a consistent and homogeneous dosimetric response. Additionally, the chemical stability of the active components—particularly their resistance to degradation from light, temperature, and humidity—is essential for ensuring accurate dose measurements and preserving the integrity of the films during long‐term storage.^73^, ^74^

The chemical and structural refinement of the active layer plays a crucial role in the performance of modern radiochromic films. A notable example is the evolution from the EBT2 and EBT3 models to the EBT‐XD film. X‐ray crystallography studies reveal that a subtle modification in the sensitive layer design of the EBT‐XD model leads to significantly smaller active particles compared to those in the EBT2 and EBT3 films (Figure 1e,f). Incorporation of significantly smaller active particles in the sensitive layer of the EBT‐XD model compared to those in the EBT2 and EBT3 films reduces light scattering and polarization effects, potentially allowing detection of dosimetric features as small as 50 µm.^10^ Consequently, EBT‐XD is well‐suited for high‐resolution applications and other small field dosimetry tasks.^9^

RCFs are considered to be water‐equivalent; meaning that the material interacts with radiation the same way we assume water interacts with radiation which is crucial since human tissue is primarily composed of water, and we are assigning dose based on this similar interaction. Most RCF models have an effective atomic number and mass density that is close to that of water (Z_eff_ ∼7.3 and density ∼1.0 g/cm^3^), allowing them to interact with radiation in a way that closely mimics water, particularly in the megavoltage photon energy range (1–25 MeV) where Compton scattering is dominant compared to photoelectric interactions. RCFs are considered water‐ or tissue‐equivalent in terms of electron density and stopping power, which makes them ideal for measuring dose distributions without introducing significant perturbations in the beam. Monte Carlo simulations and experimental validations have confirmed that RCFs, such as EBT3, exhibit dose deposition and attenuation characteristics that closely resemble those of water. This water‐equivalence makes them reliable tools for patient‐specific QA, small field dosimetry, intensity modulated radiation therapy (IMRT) and volumetric modulated arc therapy (VMAT) verification, and film‐based phantom studies.^75^, ^76^ Additionally, due to their water‐equivalent properties, RCFs cause negligible dose perturbation in the radiation field, unlike materials with higher Z_eff_ like radiographic films or metal‐based detectors. The potential for photoelectric interactions to become problematic primarily manifests at lower photon energies (≲100 keV), when increased photoelectric interactions can contribute to larger dose differences.^10^, ^11^

## 4 QUANTITATIVE FILM DOSIMETRY

RCFs are relative dosimeters; To enable quantitative dose measurements, calibration against an absolute or reference dosimeter is required. In film dosimetry, a calibration curve is established between the net OD of the films and the delivered doses. Quantitative RCF dosimetry entails three main steps: preparation, calibration, and measurement. It requires a readout device, such as a flatbed scanner, along with image analysis software capable of extracting grayscale data from the digitized film images.

### 4.1 Film handling and preparation

Proper handling of RCFs is essential to ensure accurate dosimetry. Unlike radiographic films, RCFs are less sensitive to visible light but require careful storage, handling, and cutting to prevent errors from environmental factors, surface contamination, or orientation‐dependent OD variations.

To ensure optimal dosimetric performance, RCFs should be kept in their original packaging and stored in a dark, climate‐controlled environment. Exposure to excessive heat and ultraviolet (UV) light may compromise their sensitivity and accuracy. Physical pressure, rubbing, or scratching during storage must be avoided, as even minor surface damage can significantly affect the film response. Exposure to moisture, stray radiation, and magnetic fields should also be prevented to maintain measurement accuracy and prolong the film's lifespan.^8^ Films should be stored at or below room temperature (∼22°C) and never exposed to temperatures exceeding 60°C. The recommended storage humidity is between 20% and 60%. In environments where humidity control is difficult, refrigeration is advised. When not being irradiated or scanned, films must be kept in dark envelopes or boxes to protect them from ambient light. Consistent storage conditions are essential to ensure reliable and reproducible dosimetric results.^7^

To avoid surface contamination, RCFs should be handled with clean latex or nitrile gloves, as fingerprints or smudges can alter OD readings and introduce measurement uncertainties. Care should be taken to touch only the film edges, minimizing contact with the active layer. For other vendor's RCFs, such as OrthoChromic OC‐1 film (OrthoChrome Inc., Hillsborough, NJ, USA), similar precautions are advised to prevent contamination, as per manufacturer guidelines.^77^ Films should not be subjected to mechanical stress (e.g., bending or folding), which may disrupt the monomer crystal structure and affect polymerization uniformity.

Cutting RCFs requires precision to avoid layer separation or deformation, which can occur near cut edges and lead to dosimetric errors. A sharp guillotine cutter or high‐quality scissors should be used to produce clean, straight edges without bending the film and extra mechanical stress. Specially designed punches, crafted by tool companies for specific shapes, are ideal for consistent cuts, particularly for small or complex geometries. If punches are unavailable, a minimum margin of 5 mm should be maintained between the region of interest and the film's perimeter to avoid edge effects, where layer delamination may occur. Laser cutting tools are effective for complex shapes, but the outer 1 mm of laser‐cut edges should be excluded from dosimetry due to potential thermal damage. Rectangular shapes are preferred over high‐symmetry geometries (e.g., squares, circles), as they facilitate orientation tracking and reduce alignment errors during scanning.^7^

To maintain batch consistency and traceability, individual film sections should be clearly labeled when cut from the original RCF sheet. Labels (e.g., alphanumeric codes) should be written on the film's edge outside the region of interest to avoid interfering with OD measurements. Rectangular pieces should align their long and short sides with the original sheet's dimensions to facilitate orientation tracking. Maintaining a 5 mm buffer zone around the region of interest further reduces errors from edge effects or label placement.

Based on the expected measurement dose levels, the appropriate RCF model with the desired dynamic range and sensitivity should be selected. Using a less sensitive model to measure low doses would reduce the signal‐to‐noise ratio and increase the uncertainty of the measurement. Films from the same model and same batch should be cut into small pieces (> 3×3 cm^2^) using a proper cutting device. All films should be cut along the same orientation (landscape vs. portrait, and front vs. back). The unirradiated films should be scanned under the same scanner settings (image corrections offered by the software should be turned off) to obtain their OD. To ensure that the scanner is warmed up, typically 15–25 blank scans (without the film) are sufficient to reach a stable light intensity. As the light sources may be different, it is recommended that the user assesses the stability of the light source in advance. A typical scanner configuration is 48‐bit RGB color depth (16 bits per channel), positive film mode, and a resolution of no less than 72 dpi, with all automatic image corrections turned off.

### 4.2 Film calibration

Typically, a minimum of six to eight dose (*D*) levels ((Net OD*,D*) pairs) are required to establish a reliable calibration curve. Such data points should cover the expected range of doses to be measured, with at least a couple of data points beyond the highest expected dose. Calibration films are irradiated at known dose levels, typically up to ∼ 120% of the highest expected dose level. To reduce the impact of post‐irradiation OD changes, a waiting period of 24 or 48 h prior to scanning is generally recommended. Variations in post‐irradiation waiting periods can introduce significant uncertainties and potentially necessitate rescaling of the measured dose. Using the same scanner, scanner setting, and location within the scanner, the films are scanned. Film dosimetry commercial software or in‐house scripts (MATLAB, Python, ImageJ, etc.) can be used to establish the calibration curves. The Net OD and transmittance (*T*) for each color channel, depending on the choice of calibration function, is measured over the region‐of‐interest (ROI) through

(1a) $$\begin{array}{cc} \mathrm{Net}\;\mathrm{OD} & =\log_{10}\left(\frac{PV_{0}-PV_{D}}{PV_{\mathrm{NF}}-PV_{D}}\right)-\log_{10}\\ \left(\frac{PV_{F}-PV_{D}}{PV_{\mathrm{NF}}-PV_{D}}\right) & =\log_{10}\left(\frac{PV_{0}-PV_{D}}{PV_{F}-PV_{D}}\right) \end{array}$$

(1b) $$T=\frac{PV_{F}-PV_{D}}{PV_{NF}-PV_{D}}$$

in which $PV_{0}$ is the pixel value of the unirradiated film, $PV_{NF}$ is the pixel value in the absence of any film on the scanner's bed, $PV_{D}$ is the pixel value representing the “dark current”, and $PV_{F}$ is the pixel value of the scanned irradiated film. In an “ideal” scanner, $PV_{D}$ should be zero; however, due to electronic noise and other factors, non‐zero values can be obtained when a totally opaque sample is scanned. As such, to avoid systematic uncertainties, it is recommended to subtract the background $PV_{D}$ from the measured pixel values. Typically, a totally absorbing black sheet can be scanned multiple times to obtain an average value for the $PV_{D}$ at each color channel. Ideally, $PV_{\mathrm{NF}}=2^{16}-1=65535$ for a 16‐bit‐per‐color‐channel image; however, in practice, pixel values of images without anything on the scanner's glass bed have smaller values than that.

Unfortunately, the relationship between the OD and dose is not linear. A nonlinear mathematical formula needs to be used to fit the data ((Net OD*,D*) pairs) and obtain the calibration curve. Typical formulae that have been used for calibration are^10^:

(2a) $$D=a\times NetOD+b\times NetOD^{n}$$

(2b) $$D=\frac{a.\;Net\;T}{1-b\cdot Net\;T}$$

where *a*, *b*, and *n* are fitting parameters and *T* is the transmittance.

Most commonly, Equation (2a) is used with the following associated uncertainties.^58^

(3) $$\sigma_{tot}=\sqrt{\sigma_{exp}^{2}+\sigma_{fit}^{2}}$$

in which $\sigma_{exp}$ and $\sigma_{fit}$ are experimental and fitting uncertainty obtained through the following equations.

(4) $$\sigma_{exp}\left(\%\right)=\frac{\left(a+n\cdot b\cdot Net\;OD^{n-1}\right)\sigma_{Net\;OD}}{D_{fit}}\times 100$$

(5) $$\sigma_{fit}\left(\%\right)=\frac{\sqrt{Net\;OD^{2}\cdot \sigma_{a}^{2}+Net\;OD^{2n}\cdot \sigma_{b}^{2}}}{D_{fit}}\times 100$$

where $\sigma_{Net\;OD}$ is the uncertainty associated with measurement of Net OD at each dose level obtained through Equation (6). In Equation (6), the fitting is often performed in two stages to obtain the $\sigma_{a}$ and $\sigma_{b}$ values. First, fitting is performed to determine *a*, *b*, and *n*. Then, a refined fit is obtained by fixing the *n* value obtained from the first step. In this way, smaller uncertainties are obtained for *a* and *b* parameters.^78^

(6) $$\sigma_{Net\;OD}=\frac{1}{\ln10}\sqrt{{\left(\frac{\sigma_{0}}{PV_{0}}\right)}^{2}+{\left(\frac{\sigma_{0}}{PV_{F}}\right)}^{2}}$$

Film sensitivity, defined as the rate of change of Net OD with dose, can be derived from an alternative fit model^79^:

(7) $$\mathrm{Net}\;\mathrm{OD}=A\times D+B\times D^{m}$$

(8) $$\mathrm{Sensitivity}=A+B\times m\times D^{m-1}$$

where *A*, *B*, and *M* are fitting parameters.

**FIGURE 3:**
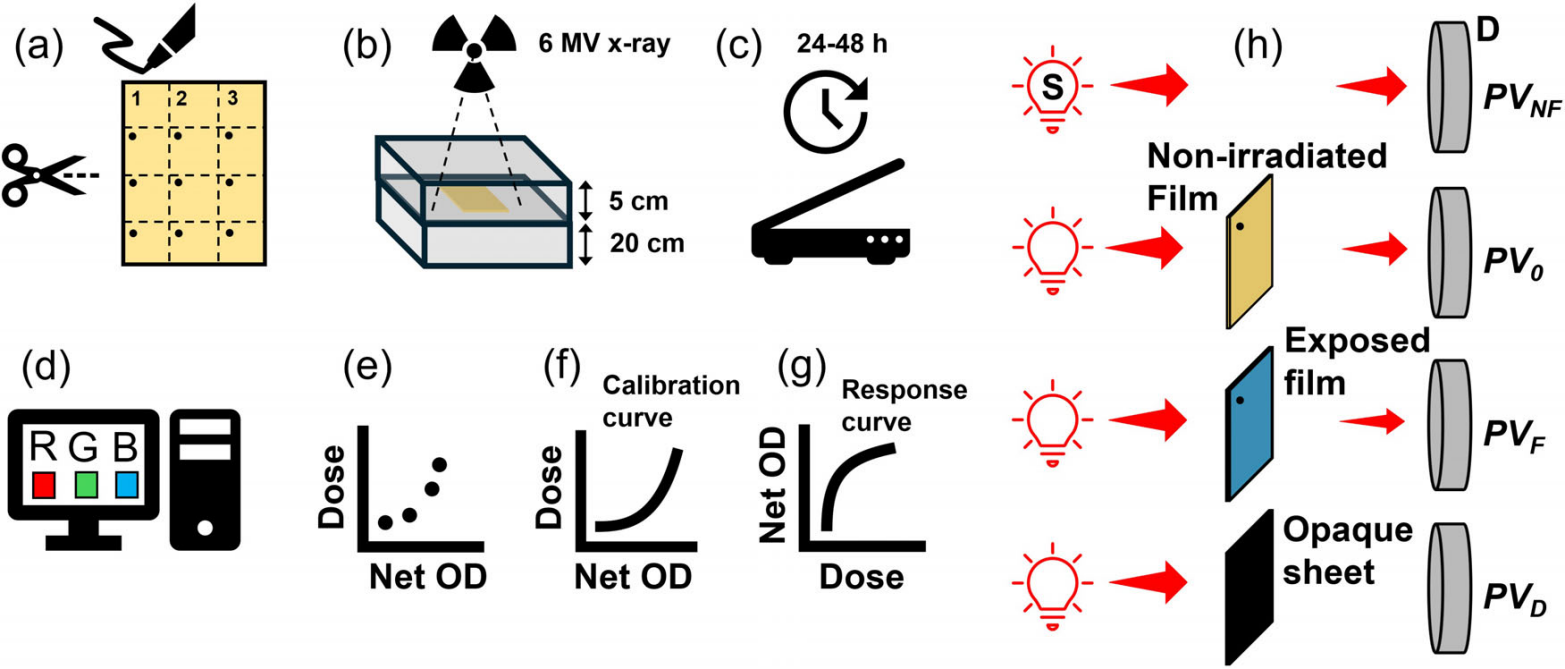
(a) Films are cut in > 3×3 cm^2^ pieces and permanently marked to ensure same orientation is maintained during pre‐ and post‐irradiation scanning. (b) Films are placed at 5 cm depth in solid water phantoms and irradiated with a 6 MV beam produced by a linear accelerator at various dose levels. (c) Using a flatbed scanner, film samples are scanned before irradiation and at a consistent time between 24‐48 hours after irradiation allowing a stable readout. (d) Using a computer script, pixel values are obtained for the red, green, and blue channels over a region‐of‐interest. (e) Net OD as a function of time for each dose level is obtained. (f) Calibration curves are obtained for a given color channel (R or G) or triple‐channel. (g) In order to calculate the film sensitivity, response curves demonstrating Net OD as a function of dose are obtained. (h) Arrangements demonstrating measurement setups to obtain *PV_NF_* (no film on the scanner's bed), *PV_0_* (un‐irradiated film), *PV_F_* (irradiated film), and *PV_D_* (an opaque sheet to obtain the dark background). S: light source; D: detector.

Figure 3 illustrates typical steps involved in film calibration. In this example, a 6 MV photon beam was used for calibration and the calibration films were placed at 5 cm depth in the phantom to avoid electron contamination and sharp dose gradients while 20 cm phantoms were placed underneath the film to provide sufficient backscatter equilibrium. Step (g) is optional to obtain the sensitivity curves for each batch.

**FIGURE 4:**
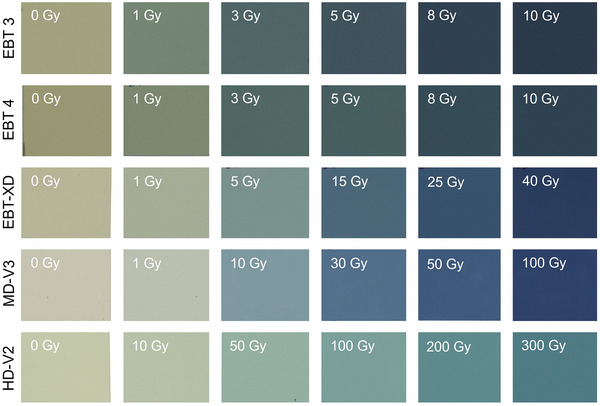
Scanned images of non‐irradiated and irradiated EBT3 (1‐10 Gy), EBT4 (1‐10 Gy), EBT‐XD (1‐40 Gy), MD‐V3 (1‐100 Gy), and HD‐V2 (10‐300 Gy) RCFs.

**FIGURE 5:**
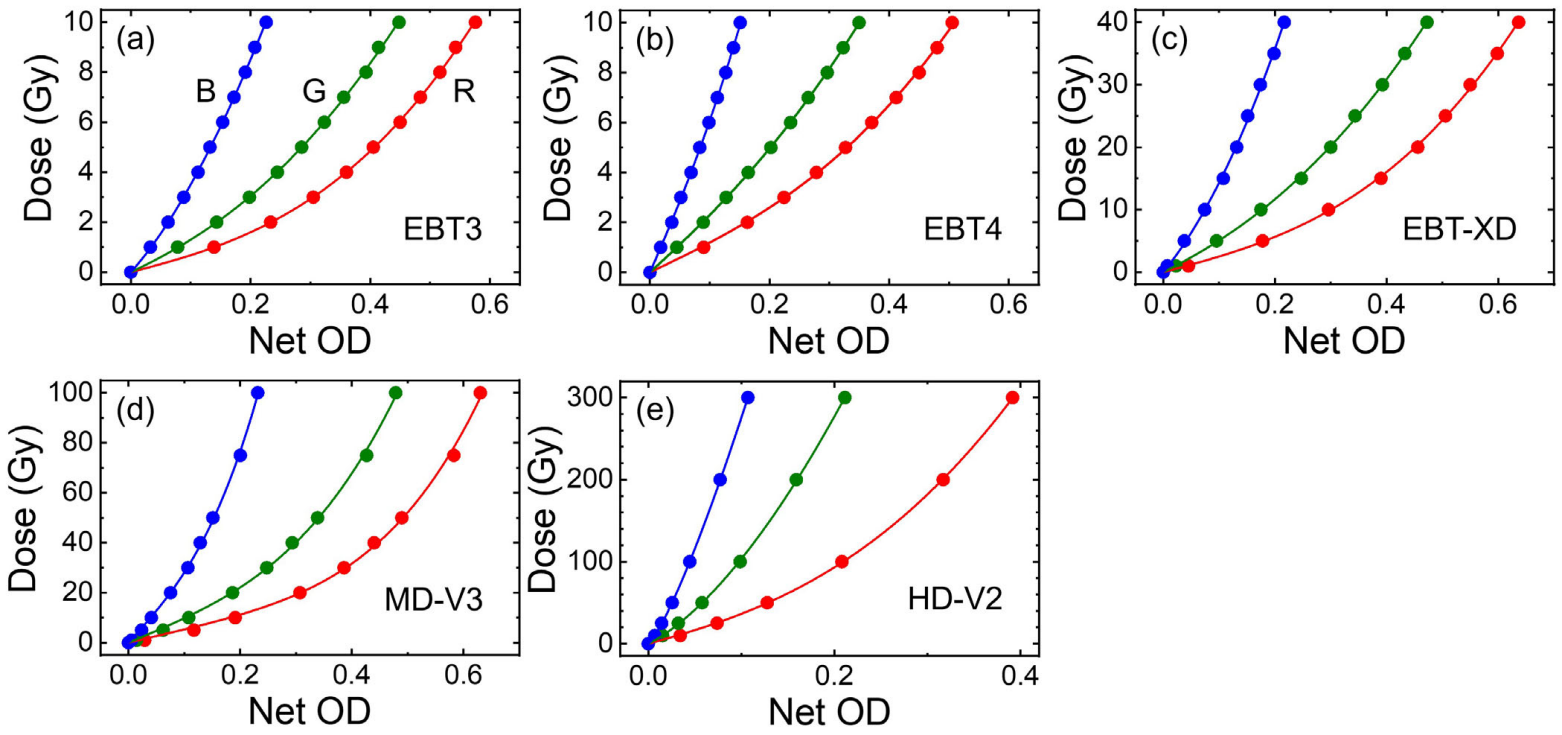
Calibration curves for red (R), green (G), and blue (B) color channels for (a) EBT3, (b) EBT4, (c) EBT‐XD, (d) MD‐V3, and (e) HD‐V2 RCFs.

Examples of EBT3, EBT4, EBT‐XD, MD‐V3, and HD‐V2 films irradiate at various dose levels are shown in Figure 4. Their corresponding calibration curves are shown in Figure 5. The calibration parameters in these examples are tabulated in Table 2.

**TABLE 2:** Example of calibration parameters for various RCF models corresponding to Figures 4 and 5. For the EBT3 model, an example is shown by using a fixed value for *n* to reduce the $\sigma_{a}$ and $\sigma_{b}$ values.

	EBT3	EBT3 (fix *n*)	EBT4	EBT‐XD	MD‐V3	HD‐V2
***a***	6.296 ± 0.443	6.296 ± 0.134	11.486 ± 0.565	23.900 ± 2.198	52.783 ± 6.422	316.32 ± 12.41
***b***	30.195 ± 1.66	30.196 ± 0.473	28.205 ± 2.954	92.396 ± 4.145	410.758 ± 61.024	2081.9 ± 124.18
***n***	2.796 ± 0.125	2.796	2.828 ± 0.233	2.899 ± 0.187	3.996 ± 0.413	2.936 ± 0.087
***R^2^***	0.9997	0.9997	0.9995	0.9995	0.9977	0.9999
***a***	11.466 ± 1.120	11.464 ± 0.293	21.996 ± 0.433	45.672 ± 3.782	94.716 ± 8.197	536.48 ± 75.68
***b***	31.764 ± 2.953	31.764 ± 1.070	32.528 ± 4.254	105.197 ± 7.871	584.159 ± 114.78	2938.98 ± 227.9
***n***	2.323 ± 0.221	2.323	2.52 ± 0.176	2.314 ± 0.206	3.244 ± 0.343	1.772 ± 0.1
***R^2^***	0.9994	0.9995	0.9999	0.9997	0.9987	0.9999
***a***	26.175 ± 2.032	26.169 ± 0.405	55.211 ± 1.328	109.998 ± 19.779	247.311 ± 15.55	71.255 ± 404.56
***b***	68.044 ± 7.748	68.023 ± 1.810	202.358 ± 111.06	373.02 ± 150.93	3371.95 ± 1504.8	4883.66 ± 225.59
***n***	1.884 ± 0.145	1.884	2.553 ± 0.349	2.05 ± 0.423	2.997 ± 0.356	1.259 ± 0.046
***R^2^***	0.9998	0.9999	0.9734	0.9989	0.9992	0.9999

**FIGURE 6:**
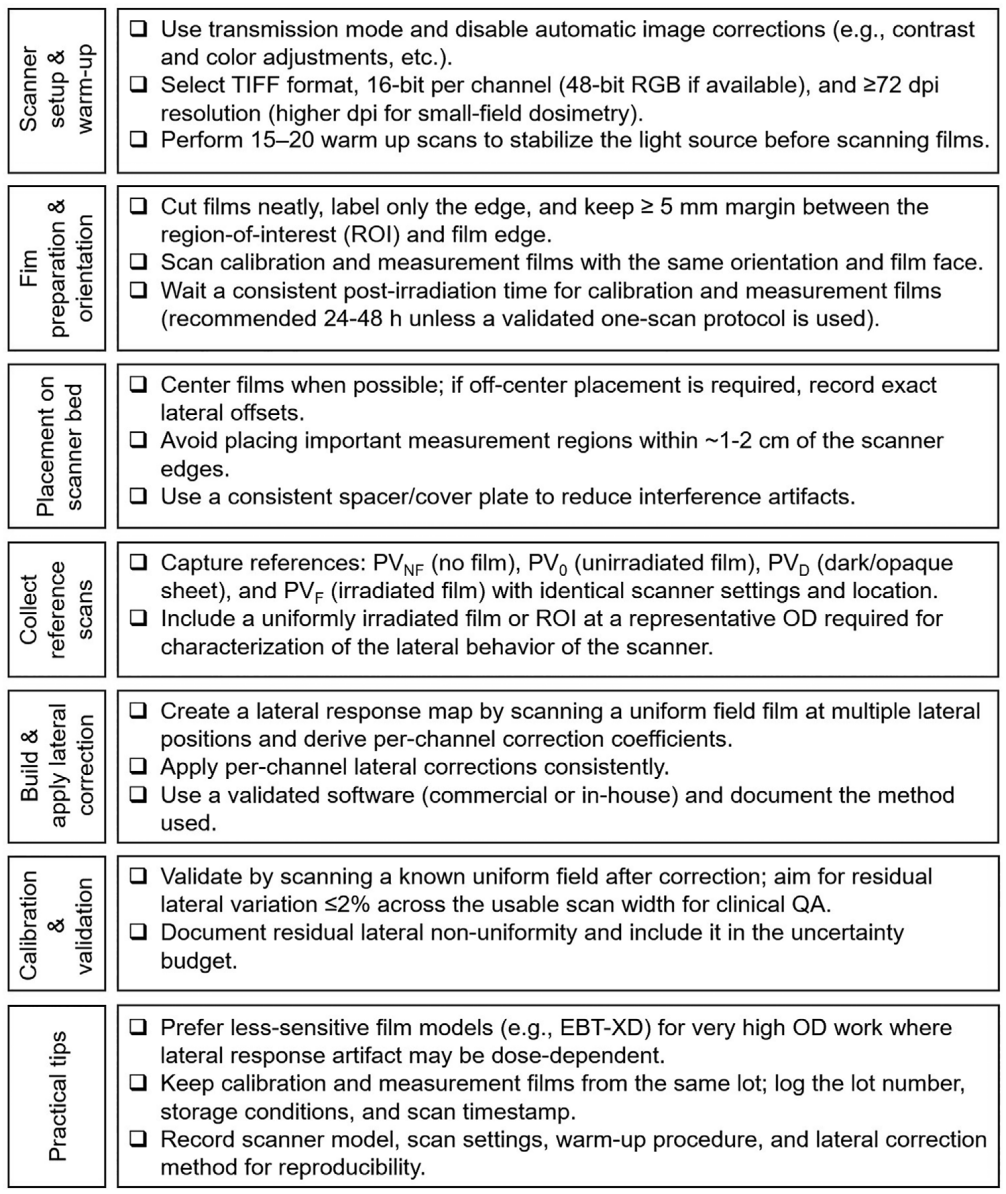
A practical checklist of steps involved in RCF dosimetry.

A practical checklist of steps involved in RCF dosimetry is provided in Figure 6.

### 4.3 Film measurement

Depending on the application, appropriate film size from the same batch and under the same orientation as the calibration films should be used for the measurement. The film is scanned prior to irradiation under the same scanner setting as the calibration films. The same post‐irradiation waiting time should be followed prior to scanning the irradiated films. A pixel‐by‐pixel analysis can be performed to obtain the dose map in 2D using Equation (2a). Ensuring high accuracy in quantitative RCF dosimetry requires strict control of environmental conditions throughout irradiation, storage, and readout, maintaining consistency at each stage.

### 4.4 Readout systems and data acquisition procedures

Upon irradiation, RCFs turn progressively darker as shown in Figure 4. which can be quantified by OD defined by Equation (1a). A calibration curve is used to define the correlation between the film's measured response and the absorbed radiation dose. This color change results from radiation‐induced solid‐state polymerization of the monomers in the film's active layer. In the EBT film series, unirradiated films appear as a semi‐transparent olive green, while irradiated films exhibit a distinct blue hue. The polymerization process not only alters the molecular structure but also flattens the film surface, contributing further to the observed color transformation. A film dosimetry system includes two main components: the RCF and a readout system. Accurate dose measurement depends on selecting the right film with suitable sensitivity for the radiation type and matching it with a readout system capable of detecting the film's color change after exposure. Efficient and high‐resolution measurement of OD or pixel grey scale value is critical, especially for large‐area films. Common readout systems include single‐point densitometers and 2D film scanners, each requiring specific film positioning techniques during acquisition. For reliable results, it is important to use calibration and measurement films from the same model and production lot and maintain consistent readout and scanning procedures.^80^ One key technical factor is the bit‐depth of the scanner. A 16‐bit scanner provides higher resolution (OD range up to $\log_{10}2^{16}=4.8$) and superior dose accuracy compared to 8‐bit mode, which may suffice for qualitative evaluation (visual inspection of radiation field, etc.) but not for precise quantitative dosimetry. A summary of various readout systems is provided in Table 3.

**TABLE 3:** Characteristics of readout systems for RCF dosimetry.

Readout System Type	Description	Advantages	Limitations
Color flatbed scanner	Uses color imaging for 2D capture in reflection or transmission mode	Widely available, captures color information	Potential artifacts
Single‐point spot densitometer	Measures OD at specific spots using a light source and detector	High precision for small areas	Time‐consuming for large areas
2D film scanner: Translational type	Scans film point by point or line by line (e.g., scanning spot densitometer, drum scanner)	High‐resolution 2D images	Slower than imaging types
2D film scanner: Imaging type	Captures entire film at once (e.g., camera‐based systems)	Fast, good for quick assessments	May have lower precision, optical distortions
2D film scanner: Combined type	Hybrid of scanning and imaging (e.g., laser scanners, VIDAR scanners)	Balances speed and accuracy	More complex, potentially expensive
Spectrophotometer	Measures absorption spectrum across wavelengths	Detailed response information, good for research	Not for routine use, complex, expensive

#### 4.4.1 Color flatbed scanner

Currently, color flatbed scanners are the most widely used readout systems in RCF dosimetry due to their accessibility, speed, and ability to capture color information, which provides a tool for dose evaluation through analysis of red, green, and blue channels. They operate in either reflection mode for opaque films or transmission mode for transparent films—the latter being more common for RCFs. Despite their convenience and speed, they may introduce artifacts like Newton's rings due to multiple reflections between the film and the glass bed of the scanner, which must be addressed through proper techniques.^81^, ^82^

#### 4.4.2 Other 2D film scanners

Several commercial 2D optical scanners are available to measure light absorption in the active layer of RCFs, using either transmission or reflection to create a digital image. Translational 2D scanners, such as scanning spot densitometers and drum scanners, measure OD across the entire film by scanning point by point or line by line. These systems produce high‐resolution 2D images (0.05–1 mm), which are invaluable for detailed dose distribution analysis in dosimetry. The scanning process, however, can be slow, particularly for large films or when high resolution is needed, making it less efficient than other methods for rapid assessments.^83^

An imaging densitometer typically includes a fixed‐position, high‐resolution 2D charge‐coupled device (CCD) camera mounted on an adjustable vertical arm, aligned above a stable light emitting diode (LED) illumination source. Both components are enclosed within a light‐tight cabinet. The RCF is placed on the lightbox surface and secured with a glass plate. The camera then captures the light transmitted through the film.^78^ This approach is significantly faster than translational scanning, making it suitable for quick evaluations or time‐sensitive applications. While it can deliver high‐resolution images, it may sacrifice some precision compared to scanning methods and is prone to optical distortions or artifacts, necessitating careful calibration and film handling.

Combined translational and imaging scanners, such as laser scanners and VIDAR scanners, integrate scanning and imaging techniques to optimize both speed and accuracy. For instance, they might use scanning for high‐precision areas and imaging for faster coverage of uniform regions. This hybrid approach offers a practical balance, though it often comes with increased complexity and cost. These systems are widely adapted from medical imaging applications, like digitizing x‐ray films, for RCF dosimetry.^74^

#### 4.4.3 Single‐point densitometers

Single‐point densitometers are among the most basic and precise tools used in RCF dosimetry. These devices measure OD at a very localized area by passing a narrow beam of light through the film and detecting how much light is transmitted.^84^ Because of their highly focused measurement, single‐point densitometers are known for their excellent accuracy, reproducibility, and minimal susceptibility to scanner‐related artifacts such as lateral response non‐uniformity. However, their major limitation lies in the fact that they only provide point‐based measurements and do not offer spatial information over large areas of the film. This makes them less ideal for applications requiring a complete two‐dimensional dose distribution, such as patient‐specific QA. Nonetheless, they have been used for calibration purposes and for precise dose verification at specific locations on the film, offering a valuable component in a comprehensive RCF dosimetry workflow.

#### 4.4.4 Spectrophotometer

A spectrophotometer measures the absorption spectrum of the film across a range of wavelengths, offering detailed insights into the film's radiation response. This makes it an excellent tool for research, such as developing new film models or refining calibration methods. However, its complexity, cost, and the need for specialized expertise limit its use in routine dosimetry, relegating it primarily to investigative or developmental purposes.^85^, ^86^

**FIGURE 7:**
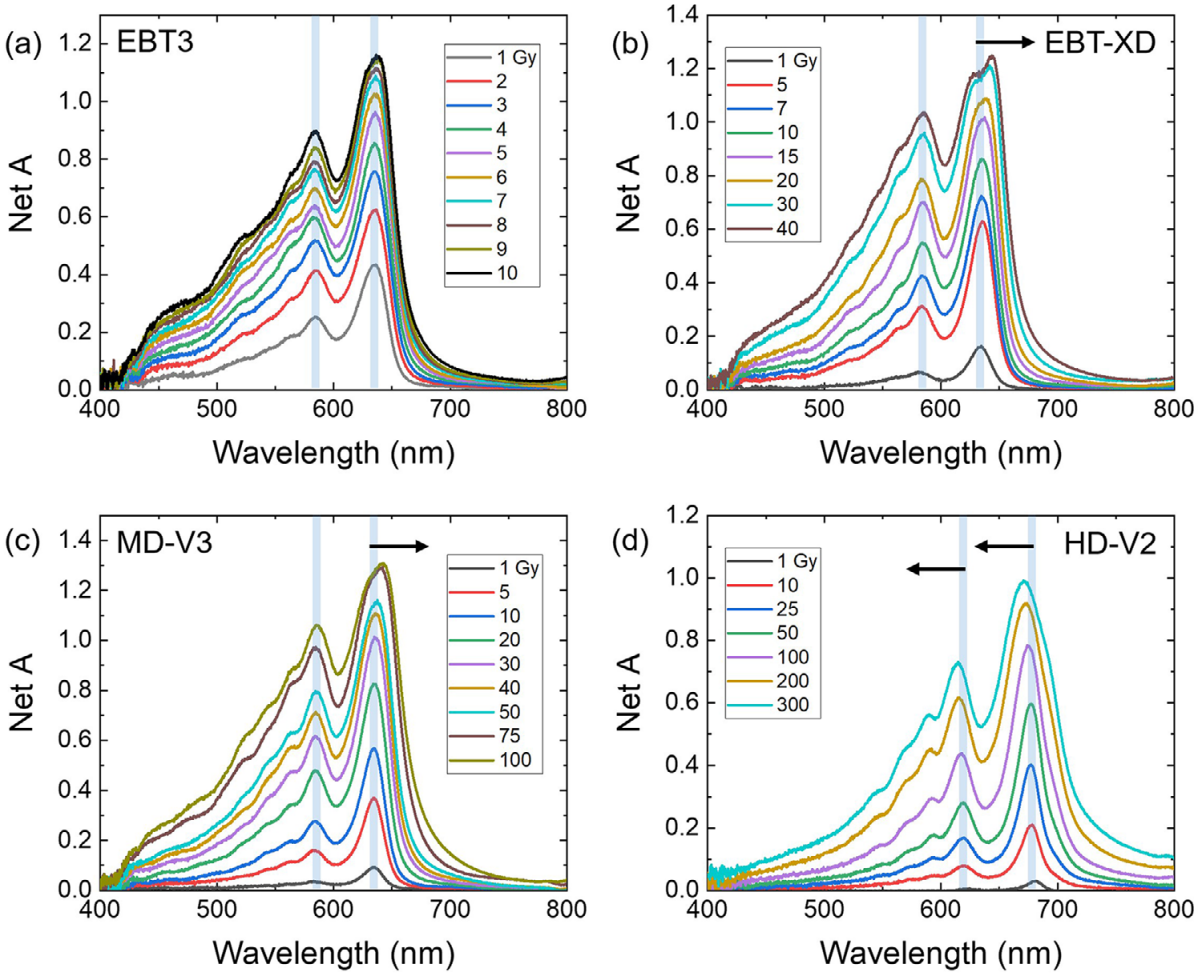
Absorption spectra of irradiated (a) EBT3, (b) EBT‐XD, (c) MD‐V3, and (d) HD‐V2 RCFs. Arrows indicated the direction of wavelength shift.^63^

Absorption spectroscopy has been performed by several investigators on various film models revealing the primary, secondary, and other absorption peaks.^65^, ^86^, ^87^, ^88^, ^89^, ^90^, ^91^, ^92^ In Figure 7, the absorption spectra for EBT3, EBT‐XD, MD‐V3, and HD‐V2 films irradiated at different dose levels are presented. The EBT3, EBT‐XD, and MD‐V3 films exhibit a dominant absorption peak near 635 nm in the red region and a secondary peak around 584 nm in the green region. For the HD‐V2 film, the main absorption peak is observed around 678 nm, with a secondary peak located near 620 nm—both within the red spectral region. However, the peak position can be changed with dose. In the HD‐V2 model, increasing the dose results in a blue shift of both the primary and secondary absorption peaks. For instance, at a dose of 300 Gy, the primary peak shifts by about 7.5 nm to 670.71 nm, while the secondary peak shifts by around 5 nm to 614.68 nm. In contrast, the MD‐V3 model exhibits a red shift in both peaks as the dose increases. Specifically, the primary peak shifts from 634.48 nm at 5 Gy to 642.43 nm at 100 Gy, while the secondary peak moves from 584.3 nm to 585.08 nm across the same dose range. The primary absorption peak shows a more noticeable shift compared to the secondary one.

The shift in absorption wavelength observed in conjugated polymers is fundamentally linked to their molecular length and structural topology.^88^, ^93^, ^94^ A well‐established relationship exists between the conjugation length and absorption wavelength—longer conjugation lengths result in absorption at longer wavelengths.^94^ In the case of EBT‐XD and MD‐V3 films, exposure to higher radiation doses induces more extensive polymerization, forming longer polymer chains. This increase in conjugation length accounts for the observed red‐shift (bathochromic shift) in their absorption spectra. Moreover, in the HD‐V2 model, the active layer is not sandwiched between two protective layers but is instead coated onto a single substrate. This reduced confinement may allow greater torsion or twisting within the polymer chains, leading to a loss of planarity. Such structural distortion effectively shortens the conjugation length, resulting in a blue‐shift in the absorption spectrum. A similar blue‐shift has been reported for the OrthoChromic OC‐1 film, which also features an active layer coated on a single base substrate without an overlying protective layer.^77^ Additionally, variations in doping levels and the chemical composition of the monomers used in the HD‐V2 film may further influence its optical behavior.

Spectral information of RCFs can be used for film calibration. One study showed that the absorption bands at 583 nm and 635 nm in EBT3 film exhibit a dose‐dependent response, with their intensity ratio serving as a reliable dose indicator.^94^ Another study demonstrated that measuring optical density across a broad spectral range (400–740 nm) for EBT3 and HD‐V2 films extends the practical dose range, especially using blue and UV wavelengths.^95^ These findings confirm that spectral analysis enhances the accuracy and versatility of RCF calibration. Spectral information of RCFs can also be utilized for film readout at high dose levels, as demonstrated by Vaiano et al. employing broadband spectral analysis of EBT3 films to accurately measure doses up to 100 Gy with less than 4% uncertainty across the range.^89^

It has been suggested that a spectroscopy setup could be used for real‐time film dosimetry. Gafchromic^TM^ EBT film has demonstrated increased sensitivity, reduced post‐exposure darkening, and stable spectral behavior throughout irradiation, further supporting its suitability for real‐time spectroscopic dosimetry applications.^95^ Recent studies have explored innovative opto‐electronic systems, such as fiber‐optic probes, to monitor radiation‐induced optical changes in RCFs during irradiation.^96^ These approaches allow for real‐time dose readout with high precision and accuracy, overcoming the limitation of traditional scanner‐based methods that only provide post‐irradiation measurements. For example, the EBT3 film has been successfully used in such systems, with results showing that its dynamic range can be extended by more than an order of magnitude.

It has been suggested that Raman spectroscopy can be used for high‐spatial resolution film dosimetry. A study demonstrated that Raman spectroscopy, combined with microscopic mapping, can detect dose‐dependent changes in the diacetylene polymerization of unlaminated EBT3 films. By measuring the intensities of characteristic Raman peaks, the technique achieved dose uniformity within ± 2% and spatial resolution on the order of a few micrometers. This approach offers a promising alternative to traditional scanning methods for precise, high‐resolution dosimetric verification.^97^

## 5 FACTORS AFFECTING THE FILM RESPONSE

### 5.1 Dose

**FIGURE 8:**
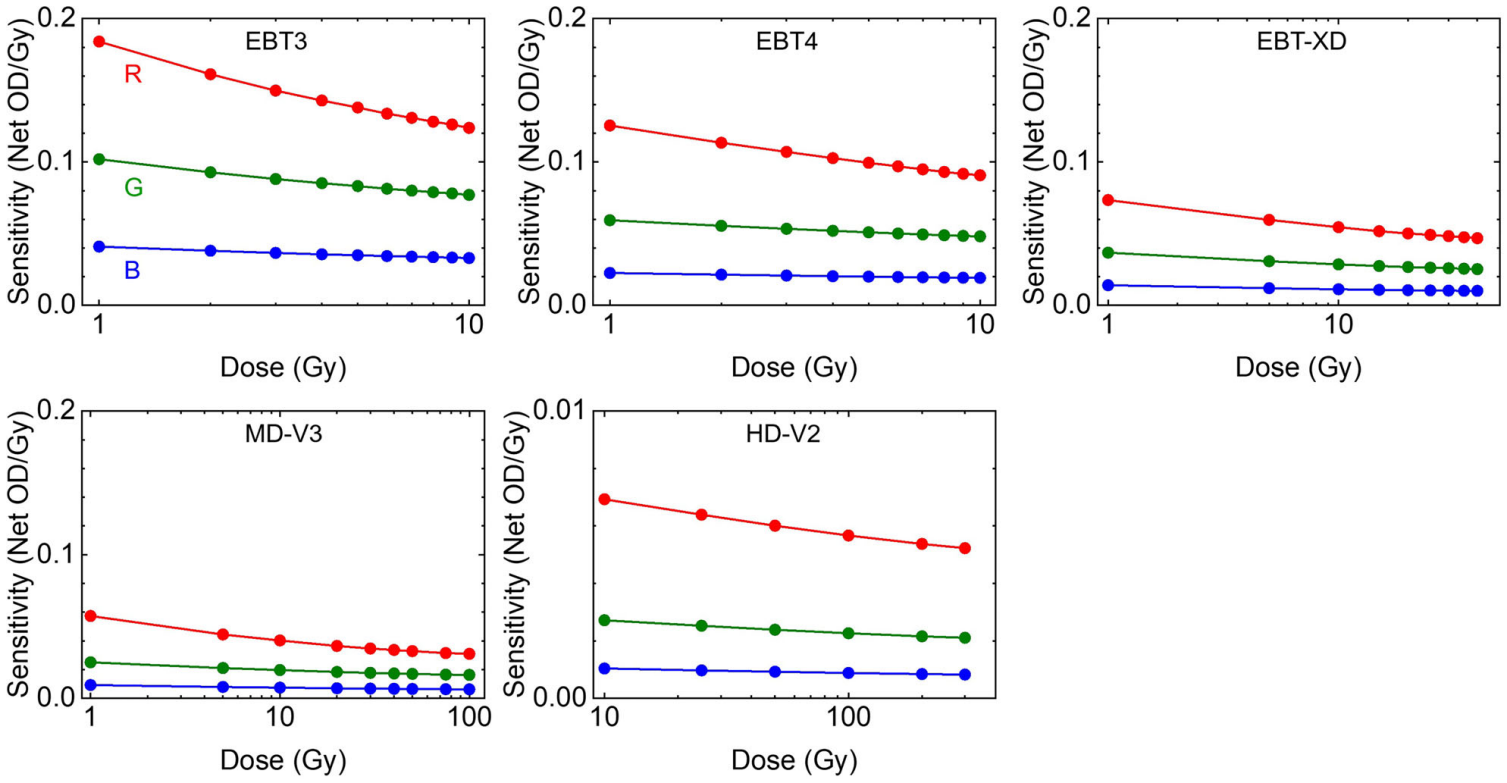
Red (R), green (G), and blue (B) color channel sensitivity plots for EBT3 (1‐10 Gy), EBT4 (1‐10 Gy), EBT‐XD (1‐40 Gy), MD‐V3 (1‐100 Gy), and HD‐V2 (1‐300 Gy) RCFs corresponding to the example shown in Figure 5.

Obviously, films’ coloration depends on the radiation dose. However, the relationship between dose and OD is not linear. Spectral response and sensitivity of the films can change with dose. The active layer's structure defines the useful dynamic range of each model for dose measurement. Due to the non‐linear relationship between OD and dose, film sensitivity reduces with dose as shown in Figure 8.

As noted earlier, the peak absorption wavelengths of films may undergo blue‐ or red‐shifts, the amount of which depends on the dose, which typically has minimal effect on film dosimetry when a broadband light source is employed to measure OD. However, when a monochromatic light source, such as a laser (e.g., HeNe with a 632.8 nm wavelength), is used for OD measurement, this dose‐dependent shift in the absorption peak position can adversely affect the OD readings.

### 5.2 Dose rate

A reliable calibration curve is essential for film dosimetry. This curve is developed during a calibration phase, where a reference beam of a specific quality and dose‐rate is employed under precise conditions. Alterations in these influencing factors can subsequently affect the dosimeter's accuracy. Dose rate dependency indicates that the detector's response (e.g., OD in RCFs) might change depending on how quickly or slowly the dose is delivered, even if the total dose received by the detector is the same. Several studies have demonstrated that modern RCFs, such as EBT, EBT2, EBT3/EBT4, and EBT‐XD exhibit minimal dose rate dependency in conventional radiation therapy settings. For example, EBT films showed a dose rate dependency of less than 1% for dose rates between 16 and 520 cGy/min.^98^ EBT2 films irradiated with flattening filter‐free (FFF) beams at 1200–2400 cGy/min dose rates showed negligible dose rate dependency, with variations within the uncertainty of scanner reproducibility and film stability.^99^ Similarly, EBT3 and EBT‐XD films irradiated by various dose rates including FFF beams showed negligible dose rate dependency.^54^, ^58^ Twork et al. reported that EBT3 films are dose rate independent within 3% across a wide range of dose rates (50–1000 Gy/min), regardless of the total dose delivered.^100^ These findings confirm the reliability of EBT‐series films for dosimetry in standard clinical dose rate ranges.

While RCFs are generally dose rate independent at higher doses, their response can vary at low doses and across different beam energies. Sharma et al. investigated EBT3 film response for MV photon beams (^60^Co, 6 MV, 10 MV, and 15 MV) over a dose range of 0.1–20 Gy.^101^ At low doses (≤1 Gy), film sensitivity (OD/Gy) deviated by up to 15% when normalized to the 6 MV beam, with dose rate effects contributing to ∼ 10.9% difference in sensitivity at 570 MU/min compared to 0.1% at 1780 MU/min. At higher doses (> 1 Gy), energy and dose rate dependencies diminished, with deviations below 5%. Additionally, film sensitivity decreased by 80%–90% as dose increased, highlighting the need for careful calibration at low doses and dose rates.

The emergence of FLASH radiation therapy, which delivers doses at rates exceeding 40 Gy/s to reduce healthy tissue damage while maintaining tumor control, has prompted investigations into RCF performance under UHDR conditions. Jaccard et al. evaluated EBT3 films using a prototype linear accelerator delivering pulsed electron beams with pulse dose rates up to 8 × 10⁶ Gy/s.^102^ The films showed no detectable dose rate dependence across mean dose rates of 0.07–3000 Gy/s and pulse dose rates of 7 × 10^3^ to 8 × 10⁶ Gy/s, with dose measurements agreeing with TLDs within 4% uncertainty for doses of 3–17 Gy. A strong linear correlation between film‐measured dose and electron pulse charge further confirmed the absence of quenching effects. In contrast, another study investigated EBT‐XD films under UHDR electron beam irradiations using a research linac with flashDiamond as the reference dosimeter.^103^ They systematically varied average dose rate, dose‐per‐pulse, and instantaneous dose rate. Results revealed an overresponse of EBT‐XD films, leading to a ∼ 10% dose overestimation at an average dose rate > 1000 Gy/s and instantaneous dose rate > 1 MGy/s compared to conventional irradiation. While EBT‐XD films offer excellent spatial resolution, caution is warranted for absolute dose measurements at extreme instantaneous dose rate values (> 1 MGy/s) in ultra‐high dose‐per‐pulse beam dosimetry. Karsch et al. assessed dose rate dependence using TLD, OSL, EBT RCFs, and diamond detectors in a superconductive linear electron accelerator generating 5 ps, 20 MeV electron pulses. Dose rates were varied by adjusting electron numbers per pulse, with TLD, OSL, and EBT films irradiated at four rates with similar doses. Results showed EBT films exhibited no dose rate dependence up to 4 × 10⁹ Gy/s within 2% and up to 15 × 10⁹ Gy/s within 5%, making them suitable for high pulse dose rate applications, such as laser‐accelerated particle beams.^104^

Villoing et al. compared EBT3, EBT‐XD, and OC‐1 films under UHDR proton irradiation (0.25–7500 Gy/s, 2–130 Gy). EBT3 and EBT‐XD films exhibited dose rate‐dependent behavior above 10 Gy, with increased Net OD at higher dose rates (up to 7500 Gy/s), potentially causing dose overestimation.^29^ OC‐1 films, however, showed no significant dose rate dependence up to 7500 Gy/s for doses above 3 Gy, suggesting greater suitability for UHDR proton dosimetry though further studies are needed for UHDR electron beams. Beam pulse structure (single vs. multiple pulses) did not affect the response of any film type. Similarly, Chen et al. reported that OC‐1 films were dose rate independent for 6 MV photon beams at clinical dose rates (2–6 Gy/min).^77^ Guan et al. assessed EBT‐XD films under therapeutic proton irradiation, including FLASH (150 Gy/s) and conventional dose rates (0.3 Gy/s).^105^ While the films showed LET dependence, with reduced response at higher LET values (1.0–9.0 keV/µm), no dose rate dependence was observed, supporting their use in FLASH proton therapy. The authors recommend a calibration approach using a specific OD calculation combined with the NIH Rodbard function for optimal fitting accuracy; further details are provided in their publication.

Overall, modern RCFs, particularly EBT3/EBT4 and EBT‐XD, are largely dose‐rate independent across standard clinical dose rates. However, in UHDR applications such as FLASH RT dosimetry, slight dependencies may emerge, particularly at low doses or extreme dose rates. These deviations should be carefully considered through appropriate calibration or correction protocols to ensure accurate dosimetry. Based on the reviewed studies, modern RCFs (EBT3/EBT4, EBT‐XD, OC‐1) can provide reliable dosimetry across UHDR regimes provided that the users validate films under matched conditions with the calibration films (same energy, pulse structure, average dose rate, dose‐per‐pulse, and LET). We recommend to (1) perform film calibrations that replicate the expected experimental pulse parameters and full dose range, including low‐ and high‐dose regions; (2) cross‐check film measurements against an independent UHDR‐validated reference detector as was done in several studies; and (3) derive specific correction factors when systematic deviations are observed.

Table 4 summarizes key studies evaluating the dose rate dependency of RCFs across photon, proton, and electron radiation modalities.

**TABLE 4:** Dose rate dependency studies for RCFs.

Dose Rate Category	Film Type	Dose Rate Range	Key Findings
Conventional	EBT^98^	0.16–5.20 Gy/min	Dose rate dependency < 1% in conventional settings.
	EBT2^99^	12–24 Gy/min	Negligible dose rate dependency, variations within scanner reproducibility and film stability uncertainties.
	EBT3^106^	0.033–4 Gy/min	EBT3 response was effectively independent of dose‐rate (within ≈3%) over a wide range of linac dose rates
	EBT3^101^	570–1780 MU/min	Less than 5% at doses over 1 Gy. Up to 15% sensitivity deviation at low doses (≤1 Gy), ∼ 10.9% at 570 MU/min vs. 0.1% at 1780 MU/min.
	EBT3^54^	2–24 Gy/min	Dose rate independent for MV photon beams.
	EBT‐XD^58^	2–24 Gy/min	Dose rate independent for MV photon beams.
	OC‐1^77^	2–6 Gy/min	Dose rate independent for MV photon beams.
UHDR/FLASH RT	EBT3, EBT‐XD, OC‐1^29^	0.25–7500 Gy/s	EBT3/EBT‐XD show dose rate dependence at > 10 Gy, increased Net OD; OC‐1 is dose rate independent at > 3 Gy, suitable for UHDR proton dosimetry.
	EBT‐XD^105^	0.3–150 Gy/s	No dose rate dependence; but LET dependence at 1–9 keV/µm; “specific OD + NIH Rodbard” calibration recommended for FLASH proton therapy.
	EBT^104^	4‐15 × 10^9^ Gy/s	EBT films exhibit minimal dose‐rate dependence, remaining within 2% accuracy up to 4×10^9^ Gy/s and within 5% up to 15×10^9^ Gy/s.
	EBT3^102^	0.07–3000 Gy/s (pulse: 7 × 10^3^–8 × 10⁶ Gy/s)	No dose rate dependence, agrees with TLDs within 4% for 3–17 Gy, no quenching effects in UHDR electron beams.
	EBT‐XD^103^	>1000 Gy/s (instantaneous dose rate > 1 MGy/s)	∼ 10% dose overestimation at average dose rate > 1000 Gy/s and instantaneous dose rate > 1 MGy/s, caution needed for absolute dosimetry in UHDR.

### 5.3 Energy

Accurate dose measurement from megavoltage radiation sources is a critical aspect of radiation therapy. However, most radiation sources also produce a portion of dose from lower‐energy scattered photons. Modern RCFs have been specifically engineered to exhibit minimal energy dependence and to closely mimic the radiation absorption properties of water and soft tissue. The response of EBT3/EBT4, EBT‐XD, and EBT4 RCFs to megavoltage photon beams, including FFF beams, has been shown to be energy‐independent or weakly energy dependent within the uncertainties of film dosimetry.^54^, ^58^ However, energy dependence has been reported when the films were irradiated with kV x‐ray beams compared to when they were irradiated with MV beams.^53^, ^84^, ^107^, ^108^

RCF models manufactured before 2014, such as HD‐810 and MD‐55, were composed exclusively of elements with atomic numbers ≤ 8 and did not contain any chemical additives. In contrast, the discontinued EBT model and pre‐2014 versions of EBT2 and EBT3 included trace amounts of chlorine‐ or bromine‐containing compounds. Starting in November 2014, all RCFs, namely HD‐V2, MD‐V3, EBT2, EBT3, EBT4, and EBT‐XD, were reformulated to include alumina within their active layers (Table IV of TG‐235^7^) to minimize their energy dependence.^67^ In addition to reducing energy dependence, the incorporation of alumina also improved their stability under varying environmental humidity conditions.

Buston et al. reported ∼ 8%–5% under‐response to kV x‐ray radiation (50–250 kVp) in EBT films when compared to MV photon beams, whereas the radiographic films showed an order of magnitude over‐response due to their high‐Z components.^108^ Under‐response in RCFs to kV x‐ray beams compared to MV x‐ray beams has been reported for other Gafchromic^TM^ film models. Eduardo and Khan reported ∼ 20% and ∼ 5% underresponse of EBT3 films to 70 kVp and 300 kVp x‐ray beam compared to ^60^Co irradiation. Massillon‐JL et al. reported ∼4%–10% underresponse in EBT3 films to 50 kVp x‐ray compared to 6 MV x‐ray irradiation.^53^ Darafsheh evaluated the energy dependence of four widely used RCF models, EBT3, EBT‐XD, MD‐V3, and HD‐V2, by comparing their OD responses to 220 kVp and 6 MV x‐ray beams.^63^ The study demonstrated that all models exhibit a measurable underresponse when exposed to kilovoltage beams, with the magnitude of the under‐response varying by dose and model; a few percent in EBT3, ∼ 5%–10% in EBT‐XD, ∼ 5%–15% in MD‐V3, and up to ∼ 25% in HD‐V2 films. In the less sensitive models, such as MD‐V3 and HD‐V2 the under‐response to kV beam was significantly reduced at higher doses reaching ∼ 2%–5% in the HD‐V2 model at doses ≥ 100 Gy. This behavior was attributed to LET‐related effects such as radical recombination and response saturation within the film's active layer. While these RCFs are designed to be approximately energy independent in the MV range, their use in kV beams requires correction or recalibration. Therefore, energy dependence must be carefully considered in low‐energy dosimetry applications, such as in orthovoltage therapy units or small animal irradiators. These results underscore the necessity of using separate calibration curves for kV and MV energy ranges to ensure accurate dosimetry, particularly in applications involving low‐energy photon beams.

### 5.4 Linear energy transfer

LET is a fundamental parameter in radiation physics that quantifies the energy deposited per unit path length of a charged particle in a material, typically expressed in keV/µm. Radiation with high LET, such as proton or heavy ion beams, deposits energy densely along its track, unlike low LET radiation (e.g., high‐energy x‐rays) which distributes energy more sparsely.^109^ In the context of RCFs, which rely on radiation‐induced polymerization to produce a measurable OD, LET plays a significant role in dosimetric accuracy. High LET radiation can lead to two primary mechanisms that reduce the OD response: (1) radical recombination, where densely generated free radicals recombine before initiating polymerization, and (2) saturation of polymerization sites, where excess ionizations overwhelm available monomers, limiting additional signal generation. These effects contribute to an underresponse in RCFs, particularly in low‐energy proton beams, such as near the Bragg peak, where LET is high.^110^, ^111^

**FIGURE 9:**
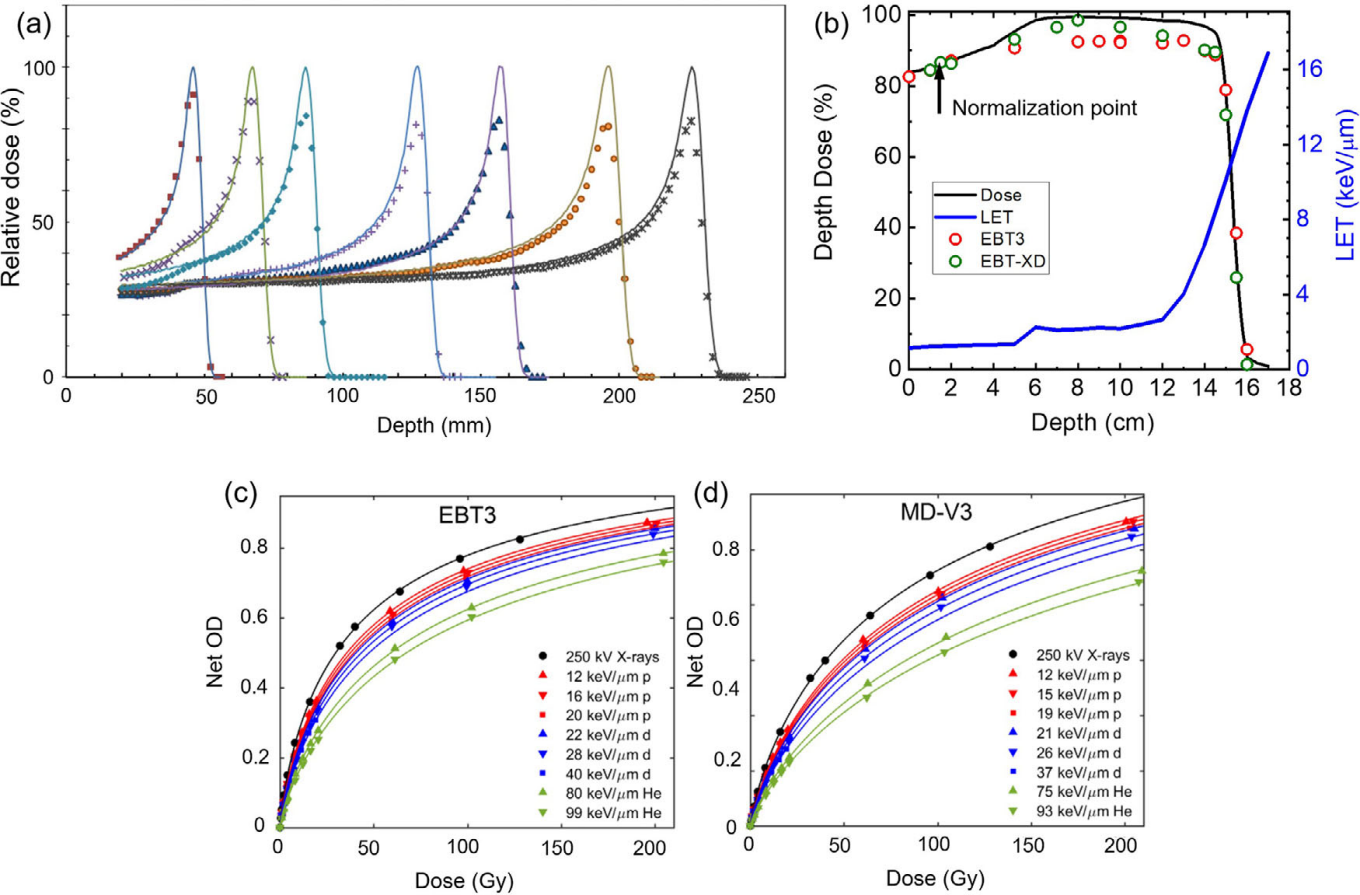
Depth dose measurement for proton (a) Pristine Bragg peaks using EBT^18^ films and (b) SOBP beams using EBT3 and EBT‐XD films.^114^, ^115^ Film quenching manifested as underestimation of the dose is evident in higher LET regions. Solid lines for dose were measured using ionization chamber. LET was calculated through Monte Carlo simulation. (c,d) LET dependency in EBT3 and MD‐V3 films.^111^

Studies have reported under‐response of RCFs to proton beams, a phenomenon referred to as “quenching”. Under‐responses of up to ∼ 20% in RCFs at low proton energies have been reported. Since LET varies as a function of depth in tissue, especially within the spread‐out Bragg peaks (SOBPs) region used in clinical proton therapy, accurate RCF‐based dosimetry demands depth‐ and energy‐specific LET correction factors.^52^, ^112^, ^113^ This is demonstrated in Figure 9a,b, where percentage depth dose (PDD) measurements using EBT and EBT‐XD films, respectively, are compared to those from an ionization chamber. After normalizing the plots at a reference depth at the plateau region, underestimation of the dose by ∼ 10%–20% can be seen in the pristine BPs. Similarly, underestimation of the dose by ∼ 5%–8% can be seen in the film measurements in the SOBP and up to ∼ 7%–8% near the distal edge, where LET is highest and quenching is most pronounced. Grilj and Brenner have studied LET dependency in unlaminated EBT3 and MD‐V3 films irradiated with ion beams with LET in the range of 12‐99 keV/µm.^111^ They reported consistently weaker OD response in the films irradiated at the same dose level with a higher LET beam (Figure 9c,d).

Correcting this quenching is complex and requires a priori knowledge of the proton beam's LET distribution, which can be obtained through Monte Carlo simulations.^116^, ^117^ The effectiveness of these correction strategies depends on the accuracy of both the simulation and the characterization of the beam configuration. Importantly, the quenching effect in RCFs has been shown to be less severe than in traditional radiographic films, making them preferable over radiographic films for high‐LET dosimetry when corrected properly.

Several LET correction models have been proposed in the literature. Zhao and Das introduced a third‐order polynomial correction based on the PDD.^18^ Kirby et al. developed a method incorporating stopping power ratios and simulated LET spectra within the film's active layer.^118^ Gambarini et al. proposed a composite correction accounting for the energy distribution within the SOBP and depth‐dependent dose deviations.^112^ Additional models include Perles et al.'s bimolecular reaction model using fluence‐weighted LET,^119^ Carnicer et al.'s exponential model,^120^ and Anderson et al.'s linear correction approach based on the LET.^121^ Building on these efforts, Resch et al. demonstrated that dose‐averaged LET (L_d_) is a significantly more reliable parameter than fluence‐averaged LET (L_t_) for correcting quenching in RCFs.^117^ Their study, supported by both experimental data and Monte Carlo simulations, revealed a strong linear correlation between L_d_ and the film's relative effectiveness. They proposed a simple and practical linear correction function that accurately restored film‐measured dose across various proton beam configurations, including monoenergetic and SOBP fields. In contrast, L_t_ failed to predict quenching in mixed‐field or modulated beams, leading the authors to recommend L_d_ as the preferred single‐parameter descriptor of beam quality for RCF dosimetry in proton therapy.

The importance of correcting for LET in proton dosimetry using RCF is clear, as many different correction methods have been proposed. However, there is still no agreement on a single best method. Because of the LET‐related effects, users must be aware of how their chosen film behaves and apply appropriate corrections for accurate dose measurement.

Although modern RCFs have been engineered to be nearly energy‐independent in the megavoltage range, measurable energy dependence appears for kilovoltage beams and for beams with substantially different LET spectra (e.g., protons/heavy ions or high‐LET regions near the Bragg peak). To ensure clinical accuracy, we recommend explicit, task‐driven recalibration rather than assuming a single universal calibration curve. It is recommended to: (1) Perform a calibration validation test when switching radiation types between calibration and measurements. (2) Perform a separate calibration when the expected error due to energy‐ or LET‐dependency of the film exceeds the expected tolerance for the given task. Since studies have reported under‐responses of several percentage up to over 10% for kV vs. MV beams, a dedicated kV calibration (or a validated spectral correction) may be required.^60^, ^122^ Since proton and ion beams exhibit LET‐quenching effects that cannot be corrected reliably by a photon‐based calibration alone; depth‐ and LET‐dependent correction or a dedicated proton calibration is required.^123^

### 5.5 Batch

Batch‐to‐batch (or lot‐to‐lot) variability in RCFs can significantly impact dosimetric accuracy, necessitating careful calibration for each film batch. Several studies have reported substantial response variations between different batches, sometimes exceeding 10% for EBT2 and EBT3 models.^52^ León‐Marroquín et al. confirmed batch‐dependent response variations in EBT3 films for clinical photon and electron beams, recommending tailored calibration protocols to account for these differences.^54^

In contrast, sheet‐to‐sheet variability within the same batch is typically minimal, with studies reporting variations of less than 1% for most RCF types, including EBT, EBT2, and EBT3. This low intra‐batch variability supports the reliability of RCFs for high‐precision dosimetry, if sheets are sourced from the same batch and handled consistently.^8^ For other film types, such as OC‐1, standardized calibration procedures are critical to achieve consistent dosimetric results across photon, electron, and proton beams, suggesting that batch‐specific calibrations remain essential to address potential variations, even if intra‐batch consistency is high.^13^ These findings collectively underscore the necessity of establishing separate calibration curves for each RCF batch, particularly for applications requiring high accuracy, such as quantitative dosimetry, to account for significant batch‐to‐batch variations while leveraging the minimal sheet‐to‐sheet variability within batches.

### 5.6 Post‐irradiation time

The temporal response of RCFs refers to the time‐dependent changes in the film's OD after irradiation. In RCFs, post‐irradiation polymerization continues to develop, affecting OD measurements over time. While the polymerization process starts quickly due to the orderly monomer arrangement, it slows down as the polymer forms, leading to a gradual increase in the OD over time. This characteristic post‐irradiation OD growth, where the rate of OD change decreases with time, can take several months to stabilize. Newer RCFs (EBT series, MD‐V3, and HD‐V2) exhibit faster post‐irradiation OD stabilization compared to earlier models (HD‐810 and MD‐55).^7^

Post‐irradiation stability of RCFs has been studied in different film models. One of the earliest studies was performed by McLaughlin et al. investigating the change in absorbance of one of the earliest models of Gafchromic™ films (DM‐1260).^124^ The films were observed to keep darkening, but the rate slowed over time; absorbance rose by about 16% within the first 24 hours after irradiation, followed by a modest increase of roughly 4% over the next two weeks.^124^ Meigooni et al. investigated the stability of both single‐layer MD‐55 and bilayer MD‐55‐2 films, reporting that their OD increased by approximately 15% during the initial 5 hours after irradiation, followed by a weekly increase of less than 3% thereafter.^125^ Therefore, it has been recommended to wait 24 h after film irradiation before performing the readout. Reinstein and Gluckman studied post‐irradiation polymerization in MD‐55‐2 films^126^, ^127^; they found slow and fast polymerization rates and reported ∼ 15% increase in the OD in the first 12 h. Ali et al. studied post‐exposure kinetics of OD in MD‐55‐2 films irradiated in 1–80 Gy range.^128^ They noted that the ratio between optical densities measured at end and start point decreased with dose. They reported a factor of ∼1.95 (1.18) increase in OD in the film irradiated at 1 Gy (80 Gy) over a 3‐month period. The MD‐55 film model has been replaced by the EBT series.

**FIGURE 10:**
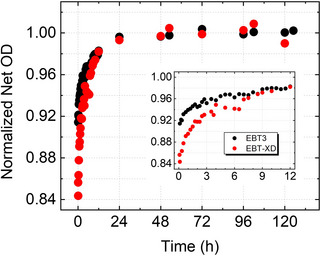
(a) Normalized OD of the red channel plotted against the readout time (hour) after irradiation at 8 Gy for EBT3 and EBT‐XD films.^115^

Zhao et al. observed an increase of approximately 7%–11% in the OD of EBT2 films within 12 h following proton beam irradiation at doses ranging from 2 to 10 Gy; they modeled the temporal behavior using a double exponential function with two time constants (T_a_ = 25 min, T_b_ = 1000 min) reflecting both rapid and slow components of polymerization.^129^ It was noted that after the first hour, the change in OD per 10 minutes was less than 1%. Borca et al. reported ∼ 2.4%–4.3% increase in the OD between 1 h to 24 h post‐irradiation in EBT3 films irradiated by 6 MV photon beams (0.1–7 Gy)^51^; between 2 h and 24 h post‐irradiation time, the OD increased by only ∼ 2.5% in all dose levels. The post‐irradiation darkening in EBT3 and EBT‐XD film models was reported by Darafsheh et al.^115^, ^130^ At 8 Gy dose, they reported an ∼ 7% and 15% increase in the OD within the first 24 h post‐irradiation in EBT3 and EBT‐XD films, respectively (Figure 10); after 24 h, the OD reached a steady state. The difference between the two models is associated with the structural differences between the two film models allowing different dynamic ranges (0.1–10 Gy in EBT3 and 0.4‐40 in EBT‐XD); EBT‐XD being less sensitive, allows more polymerization to occur over time compared to the EBT3 model. Recently, Liu et al.^131^, Dunn et al.^132^, and Caprioli et al.^133^ have studied post‐irradiation darkening in EBT3 films to propose a time‐independent film dosimetry protocol that can take into account the temporal growth in the OD of the films post‐irradiation.

Chen et al. studied the temporal growth of the OD in OC‐1 films ^77^; compared to the Gafchromic^TM^ films, the rate of change was higher in the OC‐1 model. Approximately 30% (15%) increase in the Net OD of the red color channel was reported during the first 48 h post‐irradiation for the film irradiated with 10 Gy (50 Gy). OC‐1 films reached ∼ 80% of final OD within 4 h post‐irradiation across photon, electron, and proton beams, with slower growth up to 48 h. Within the first 48 h, Net OD increased by approximately 30%–33% (10 Gy) and 15%–19% (50 Gy). Over a 90‐day period, the total Net OD change reached around 74%, 86%, and 88% in the red, green, and blue channels respectively for the 10 Gy dose, and 33%, 42%, and 45% for the 50 Gy dose. These results highlight the importance of consistent scanning timing for calibration and measurement films. The temporal changes were accurately modeled using both double‐exponential (Equation 9) and triple‐exponential (Equation 10) functions, with the triple‐exponential model yielding better fits and lower errors (∼ 1%) for the red and green channels. This confirms the need to account for time‐dependent changes in OD for precise dosimetry using films.

(9) $$Net\;\left(OD\right)=Net\;OD_{Final}-\left(C_{1}e^{-\frac{t}{T_{1}}}+C_{2}e^{-\frac{t}{T_{2}}}\right)$$

(10) $$Net\;\left(OD\right)=Net\;OD_{Final}-\left(C_{1}e^{-\frac{t}{T_{1}}}+C_{2}e^{-\frac{t}{T_{2}}}+C_{3}e^{-\frac{t}{T_{3}}}\right)$$

In the above equations, *t* is the post‐irradiation scanning time, $Net\;OD_{Final}$ is the latest readout of the OD of the films and $C_{1},C_{2},C_{3},T_{1},T_{2},$ and $T_{3}$ are different fitting parameters in each equation.

To achieve accurate dosimetry, it is recommended that the same post‐irradiation reading time should be followed for the calibration and measurement films. Since the rate of change in OD is very slow after 24 h post‐irradiation (see Figure 10), typically it is recommended to wait at least 24 h (or conservatively, 48 h) before scanning the films. Otherwise, a correction should be applied if scanning time for the calibration films is different from that for the measurement films.^134^ To address temporal response challenges in time‐sensitive applications, techniques such as scanning calibration and measurement films within 30 min or using a “one‐scan” protocol with embedded dose corrections can reduce waiting times, though they may introduce higher uncertainties.^36^, ^128^ Environmental factors, such as temperature and humidity, can also influence polymerization rates, necessitating consistent storage conditions post‐irradiation to minimize variability. These findings underscore the importance of film‐specific scanning protocols and environmental controls to optimize dosimetric accuracy across diverse RCF types and applications.

### 5.7 Orientation

Film orientation refers to the positioning or direction of a RCFs relative to the readout system's (e.g., scanner) light source during OD measurement (e.g., film scanning). The elongated nanocrystals in the active layer of the films mimic a polarizer's behavior. The needle‐shaped monomers in the active layer can modulate the polarization of the incident light which is manifested as an orientation dependency when the OD is measured using a polarized light source or polarization sensitive detector.^81^ Several investigations have shown orientation dependency of the now obsolete older generations of RCFs, such as MD‐55,^135^ HS,^136^ EBT,^137^ and EBT2^82^, ^138^ models, as well as existing XRQA,^139^ EBT3 and EBT‐XD^56^, ^57^ models. Since the optical properties of RCFs depend on the polarization of the incident light, scanning the film in different orientations can cause measurable differences in the OD or transmittance of the films.^8^, ^10^ Two common types of orientation errors are (1) landscape vs. portrait (scanning the film horizontally versus vertically with respect to the film's sheet), and (2) front side vs. back side (scanning with the active layer facing up or down, which is especially important for films with asymmetrical structures).

Inconsistent orientation between calibration and measurement films can introduce systematic errors of up to several percentages, depending on the film type and scanner. To minimize uncertainty, all films should be scanned in the same orientation. Either portrait or landscape is acceptable, but they must not be mixed within the same dataset. Some RCF models are asymmetrical by design, including Type 1 and Type 2 configurations, and Type 3 if Substrate #1 and Substrate #2 differ in thickness. In such cases, the same side must face up during irradiation and pre‐ and post‐irradiation scanning to ensure consistency.^7^, ^10^

**FIGURE 11:**
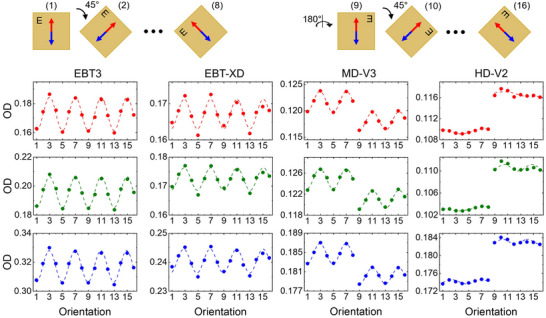
The OD's dependence on orientation measured across R, G, and B color channels for unirradiated EBT3, EBT‐XD, MD‐V3, and HD‐V2 films. Dashed lines represent cosinusoidal squared curve fits. For MD‐V3 and HD‐V2 films, orientations 1–8 and 9–16 were fitted separately. Note that the substrate layers are asymmetric in MD‐V3 as such the response changes depending on which side of the film faces the scanner. In HD‐V2 films with a single layer, the direction dependency is more pronounced. The inset shows how the orientations were defined. The arrows indicate a hypothetical preferential polarization direction in the films.^63^

RCF monomer crystals are needle‐shaped and exhibit polarizing behavior akin to a linear polarizer. Therefore, films scanned with polarized light sources (e.g., laser or LED) are particularly sensitive to orientation. To avoid artifacts, it is essential to label the cut film strips and maintain consistent orientation during calibration, background scanning, and measurements.^8^ As described by Malus's law, light transmitted through a linear polarizer follows a cosine‐squared dependence, $I=I_{0}\cos^{2}\theta$, in which I is the transmitted intensity, $I_{0}$ is the initial intensity, and θ is the angle between the polarization axis of the beam and the polarization axis of the linear polarizer.^63^ A recent study investigated the orientation dependency of EBT3, EBT‐XD, MD‐V3, and HD‐V2 films by scanning un‐irradiated samples in 16 orientations using a flatbed scanner.^63^ All models exhibited cosinusoidal OD variations, modeled by Malus's law, indicating a preferential polarization axis driven by needle‐shaped monomer crystals in the active layer. EBT3 films showed the largest OD changes, up to 16% in the red channel, 13% in green, and 8% in blue across orientations. EBT‐XD films had smaller variations (7% red, 6% green, 4.3% blue), while MD‐V3 and HD‐V2 showed 6%–6.4% (red) and 7.8%–8.6% (red), respectively. For same‐face scans, variations were reduced in the MD‐V3 and HD‐V2 models. It was 16% (EBT3), 7% (EBT‐XD), 3.3% (MD‐V3), and 1.3% (HD‐V2) in the red channel. Figure 11 shows orientation dependency of EBT3, XD, MD‐V3, and HD‐V2 films. The study highlighted that films with longer monomer crystals (e.g., EBT3) exhibit stronger orientation dependency due to enhanced polarizability, while smaller crystals (e.g., HD‐V2) reduce this effect. Asymmetric designs in MD‐V3 (adhesive layer on one side) and HD‐V2 (active layer on a single substrate) caused additional OD changes when the film was flipped, emphasizing the need to maintain consistent film side orientation.^63^ A similar polarization‐driven behavior was noted for OC‐1 films, which, like HD‐V2, have an active layer coated on a single substrate contributing to orientation effects.^77^ To mitigate orientation dependency, using an unpolarized light source for scanning has been suggested, as it minimizes polarization modulation.^91^, ^140^

### 5.8 Lateral response artifact

The lateral response artifact refers to a systematic deviation in OD measurements across the lateral (x‐axis) direction of a scanned RCF.^141^ Even if the dose is uniform across the film, the scanner may record varying pixel values depending on the position, typically showing lower OD values near the edges of the scanner bed compared to the center. For example, EBT3 film's response varies minimally near the scanner's center but increases significantly toward the lateral edges, especially at higher doses and in the red color channel. Multi‐channel dosimetry (any method that uses more than one color to determine dose from a scanned RCF image) may reduce this effect, but errors can occur if films are not centered, if exposed areas reach the scan window's edges, or at very high doses (> 1000 cGy).^66^ LRA mainly arises from two factors: light scattering within the film and polarization effects from the scanner's optical system.^142^ Less light reaches the detector at the film edges due to angle‐dependent mirror reflectivity in the scanner, and this effect worsens as the film darkens with dose. Consequently, the artifact depends on both the film's position on the scanner and the radiation dose.^143^

Lewis and Chan proposed a practical correction method to eliminate the LRA in EBT3 RCF dosimetry caused by scanner nonuniformity.^143^ They measured film responses at multiple lateral positions across the scanner bed and derived correction coefficients (AL,X and BL,X) for each color channel. These coefficients allowed them to mathematically adjust film responses from any lateral position to match the scanner's center. This correction was validated across multiple film lots, scanner models, and clinical field types (e.g., IMRT, VMAT), significantly improving dosimetric accuracy and preserving compatibility with triple‐channel dosimetry. LRA has been examined in EBT‐XD film and compared to EBT3 and it has been found that both films exhibit LRA behavior due to scanner optics^144^; however, the impact of LRA is reduced for EBT‐XD due to their decreased sensitivity, especially in high‐dose applications (> 10 Gy). The study also confirmed that changes in the crystalline structure between EBT3 and EBT‐XD do not eliminate LRA, but EBT‐XD is still preferable for high‐dose measurements due to its reduced artifact influence and steeper red/green channel response curves. Recently, Miura et al. evaluated the scanning orientation and LRA in EBT4 films and compared them to EBT3.^145^ They found that EBT4 showed improved performance regarding both LRA and scanning orientation effects. At 2 Gy, the OD differences due to orientation were lower in EBT4 (3.9%) compared to EBT3 (6.8%), and dose deviations due to LRA at 10 cm off‐center were slightly reduced (13% in EBT4 vs. 16% in EBT3). Overall, EBT4 exhibited better stability and less sensitivity to scan position and orientation, making it a refined option compared to the EBT3 model for reducing LRA‐related uncertainties in film dosimetry.

**FIGURE 12:**
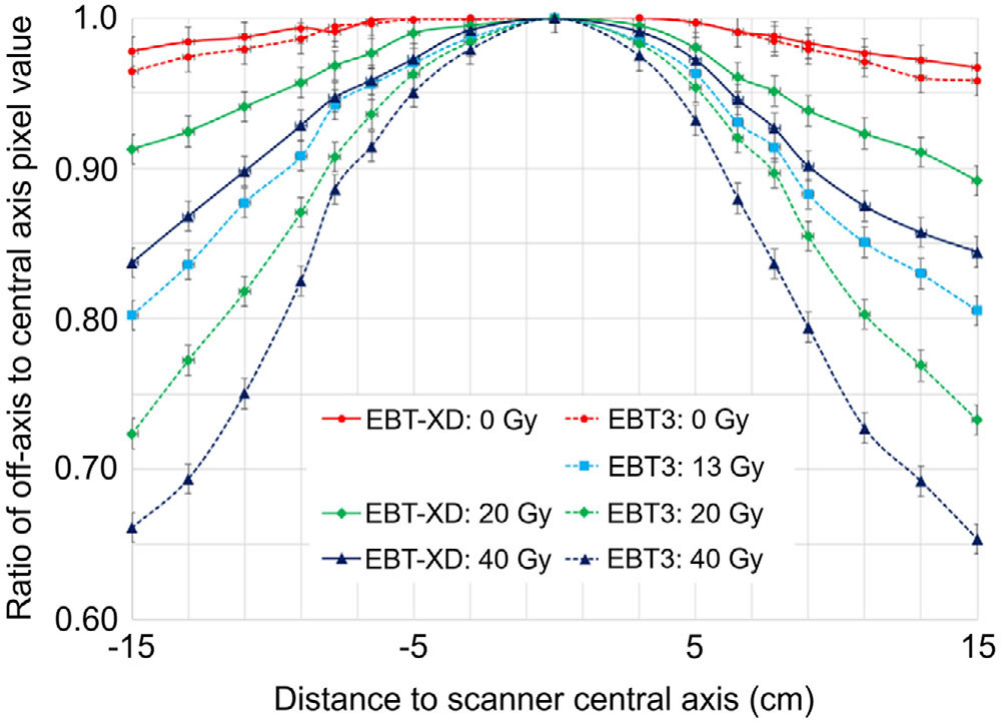
Lateral response artifact observed in EBT‐XD and EBT3 films over the dose range of 0–40 Gy scanned by an Epson 11000XL scanner.^146^

Figure 12 illustrates the LRA in EBT‐XD and EBT3 films by showing the relative change in pixel value across the width of an Epson 11000XL scanner for doses of 0, 20, and 40 Gy, with an additional 13 Gy film for EBT3 selected to match the OD of the 40 Gy EBT‐XD film.^146^ The data demonstrate that EBT3 films exhibit a more pronounced lateral effect than the EBT‐XD films, especially at higher doses. At 40 Gy, the pixel value in EBT3 film drops by up to 35% at 15 cm from the central axis, whereas EBT‐XD shows significantly lower variation. Even when the ODs are matched, EBT‐XD films experience less lateral distortion, making them more suitable for high‐dose applications such as SRS, where the lateral effect remains within 0.5% for EBT‐XD and 2.0% for EBT3 over a 3 cm field.

### 5.9 Temperature and humidity

The impact of temperature on the OD of RCFs has been thoroughly investigated.^73^ Raising the film temperature from 22°C to 38°C causes a linear variation in absorbance, primarily due to changes in the hydration levels of the film's active materials. These changes alter the position of the absorbance peak and impact the overall sensitivity of the film. Additionally, humidity has been shown to introduce systematic dose errors in RCF dosimetry.^147^ Variations in humidity from 80% to 20% can result in dose errors of up to 15%, emphasizing the need for strict control over film storage conditions, particularly temperature and humidity.

León‐Marroquín et al. investigated the effects of humidity by examining water immersion of EBT3 RCFs. Their findings revealed that water infiltration at the film edges, up to ∼4.2 mm after 24 h, led to increased Net OD in the central region, even in areas not directly penetrated by water.^148^ This effect was attributed to chemical interactions between water and the cover layer of the film, resulting in up to a 16.8% increase in net OD at a dose of 3 Gy after just one hour of immersion. These results align with previous reports indicating humidity‐induced dose errors as high as 15% when humidity varies from 80% to 20%.^147^ In another study, Trivedi et al. explored the combined effects of temperature and humidity on EBT3 film performance. Films stored at 40°C for 48 h exhibited a mean relative sensitivity of 1.107 ± 0.123, leading to a 7.01% dose overestimation across the 2–20 Gy range. Conversely, films stored at 2°C demonstrated a relative sensitivity of 0.966 ± 0.028, corresponding to a 4.54% dose underestimation when compared to films stored at 20°C.^149^ Regarding humidity, water submersion caused a diffusion rate of 0.23 mm/h at the film edges and resulted in a 6.8% increase in the OD at the central region of unexposed films after 24 h. These findings underscore the importance of controlling storage temperature and humidity and implementing corrections for Net OD changes, especially when films are submerged in water for extended periods (e.g., beyond 4 h), to ensure accurate RCF dosimetry.^149^

For optimal performance, RCFs need to be kept in a dark, temperature‐regulated setting, preferably in their original packaging. Storage temperatures should be at or below room temperature (around 22°C), and films must never be subjected to temperatures above 60°C. The recommended storage humidity is between 20% and 60%. In environments where humidity control is difficult, refrigeration is advised.

### 5.10 Mechanical stress and light

RCFs are sensitive to mechanical stress and UV light, which can degrade their dosimetric properties. Physical pressure, rubbing, bending, or scratching during storage or handling must be avoided, as even minor surface damage can significantly affect the film response. Consistent storage conditions are essential to ensure reliable and reproducible dosimetric results. When not being irradiated or scanned, films must be kept in dark envelopes or boxes to protect them from ambient light. Exposure to moisture, stray radiation, and magnetic fields should also be prevented to maintain measurement accuracy and prolong the film's lifespan.

### 5.11 External magnetic field

MRIgRT employs real‐time MRI to visualize anatomy during radiation delivery, necessitating dosimeters that perform reliably in external magnetic fields of 0.35–1.5 T.^150^ While these fields do not alter primary photon beams, they influence secondary electron trajectories via the Lorentz force, potentially affecting dose deposition and dosimeter response.^151^, ^152^ The magnetic field affects electron paths, thereby altering the dose deposited in the medium, which can also partially influence the response of commonly used dosimeters. RCFs have been studied in the context of MRIgRT dosimetry, as summarized in Table 5 across photon and proton beams. Common consensus is that the impact of the external magnetic field on RCFs is within the overall uncertainty of film dosimetry.

**TABLE 5:** Summary of film dosimetry literature in the context of MRIgRT.

Film model	Clinical MRIgRT	Concurrent B‐field and irradiation	Magnet	B‐field (T)	Radiation type (/ Reference)	Dose range (Gy)	Result
EBT2	No	No	MRI scanner (GE)	1.5	6 MV	0–8	∼ 4% under‐estimation of dose.^153^
EBT2	Yes	Yes	MRIdian (Viewray)	0.35	^60^Co (/ Same system with magnet off)	2–18	8.7%, 8%, and 4.3% reduction in the Net OD for red, green, and blue channels, respectively.^154^
EBT3	No	Yes	Electromagnet	1.5	^60^Co	2–8	< 2% under‐response, but it was within the measurement uncertainty.^155^
EBT3	No	Yes	Electromagnet	0.35‐1.42	6 MV	1–20	Up to 2.1% under‐estimation of dose.^22^
EBT3	Yes	Yes	MRIdian (Viewray)	0.35	^60^Co (/ Linac for B = 0)	1–8	Within 1% uncertainty of the measurements. And 1.5% when real‐time MRI was performed.^156^
EBT3	No	Yes	Electromagnet	0.5 ‐ 1	62.4 ‐252.6 MeV Proton	0.2–10	No significant change in film response.^157^
EBT3	No	Yes	Electromagnet	0.5 ‐ 2	^60^Co	0.75–8	Within measurement uncertainty for B ≤ 1.5 T. But, 2.4% over‐response at B = 2 T.^158^
EBT2, EBT3, EBT‐XD	No	No	MRI scanner (Siemens)	1.5 ‐ 3	6 MV & 6 MV FFF	0–20	No significant change in film OD.^159^
EBT3, EBT‐XD	Yes	Yes	MRIdian (Viewray)	0.35	6 MV FFF (/ Linac for B = 0)	0–20	No significant change in film response.^23^

For 6 MV photon beams, Reyhan et al. reported ∼ 4% dose under‐response in EBT2 films exposed to 0–8 Gy in a 1.5 T MRI scanner.^153^ Under a 0.35 T MRIdian system (2–18 Gy), Reynoso et al. reported net OD reductions of 8.7%, 8%, and 4.3% in red, green, and blue channels, respectively, in EBT2 films compared to a zero‐field setup.^154^ Delfs et al studied irradiated EBT3 films with 1–20 Gy in 0.35–1.42 T electromagnets and reported up to 2.1% dose underestimation, within measurement uncertainty.^22^ Under a 0.35 T MRIdian system with 6 MV flattening filter‐free (FFF) beams (0–20 Gy), Darafsheh et al. reported no significant response changes in EBT3 and EBT‐XD films compared to a zero‐field linac, supporting their reliability for clinical MRIgRT.^23^ Similarly, EBT2, EBT3, and EBT‐XD films tested in 1.5–3 T MRI scanners with 6 MV and 6 MV FFF beams (0–20 Gy) displayed no notable OD changes, reinforcing their robustness up to 1.5 T.^159^ EBT3 films in a 0.35 T MRIdian system (1–8 Gy) showed responses within 1% uncertainty, increasing to 1.5% with real‐time MRI, suggesting high reliability.^156^ At 1.5 T with ^60^Co (2–8 Gy), EBT3 films displayed a < 2% under‐response, within uncertainty.^155^ Another study at 0.5–2 T with ^60^Co (0.75–8 Gy) found EBT3 responses within uncertainty for B ≤ 1.5 T, but a 2.4% over‐response at 2 T, indicating a potential threshold for magnetic field effects.^158^ For proton beams, limited data suggest EBT3 films are unaffected by magnetic fields. In 0.5–1 T electromagnets with 62.4–252.6 MeV protons (0.2–10 Gy), EBT3 films showed no significant response changes, supporting their use in proton MRIgRT.^157^

## 6 UNCERTAINTY BUDGET

The uncertainty budget for RCF dosimetry quantifies the contributions of various factors to the overall uncertainty in dose measurements. These factors include film characteristics, irradiation conditions, readout systems, and data processing methods, as outlined in the AAPM's Task Group 235 report.^7^ The total uncertainty depends not only on the RCF dosimetry system but also on the accuracy of the radiation delivery system and measurement protocols.

### 6.1 Film‐related uncertainties

The film‐related uncertainties in Table 6 account for variations inherent to the RCF's properties and handling. The lot‐to‐lot sensitivity variation is estimated at 2.9%, reflecting differences in sensitivity between film batches due to manufacturing variations. The TG‐235 report notes a typical ± 5% variation, which, when converted to a standard uncertainty using a rectangular distribution (5%÷√3), yields approximately 2.9%. This is supported by studies like Devic et al. which highlight the importance of using same‐lot films to minimize this effect.^10^ Film non‐uniformity contributes 1.5%, arising from variations in the active layer's thickness or composition. Modern films like EBT3 and EBT‐XD have improved uniformity, with TG‐235 reporting a 1%–2% range, and Battum et al. confirm similar values, making 1.5% a reasonable estimate.^142^ Post‐irradiation OD growth is assigned at 2.0%, as the film's OD continues to develop post‐irradiation, stabilizing after ∼24 h. TG‐235 suggests a 1%–3% uncertainty if scanned prematurely, and Martišıkova et al. note that waiting 24 h keeps this around 1%–2%, supporting the 2.0% average.^160^ Finally, film handling and storage contributes 0.8%, reflecting minor effects from improper handling (e.g., light exposure) or storage conditions (e.g., temperature variations). TG‐235 (Section 6.D.1) estimates 0.5%–1%, and controlled conditions, as emphasized by Devic et al. justify the 0.8% value.^10^

**TABLE 6:** Uncertainty budget in RCF dosimetry.

Category	Source of Uncertainty	Estimated Uncertainty (%)	Typical uncertainty range (%)	Practical mitigation / best practices
Film‐Related Uncertainties	Lot‐to‐lot sensitivity variation	2.9	1–5	Use same‐lot films; produce a calibration curve per lot; keep manufacturer lot certificate; use reference films and include lot information in reports.
	Film non‐uniformity	1.5	1–2	Avoid edge regions; map uniformity (flat‐field exposures) and apply corrections or exclude non‐uniform regions.
	Post‐irradiation OD growth	2.0	1–3	Standardize and report time between irradiation and scan (e.g., 24 h); if scanning earlier, use time‐dependent correction factors.
	Film handling and storage	0.8	0.5–1	Store in lightproof sleeves, controlled temperature/humidity; handle with gloves; avoid creasing; keep records of storage conditions.
Irradiation‐Related Uncertainties	Dose delivery accuracy	1.5	1–2	Maintain linac QA traceable to standards (ion chamber/ADCL); perform cross checks with independent detectors before critical measurements.
	Setup variability	1.5	1–2	Use indexed/rigid phantoms, laser alignment, imaging verification; document setup and repeat measurements.
	Energy dependence	2.0	1–3	Use energy‐specific calibrations or correction factors; avoid using a single MV calibration for kV/brachytherapy without validation.
Readout System Uncertainties	Scanner variability	1.5	1–2	Use the same well‐characterized scanner; warm‐up scans; perform routine scanner QA; avoid large scanning area without correction.
	Scanner calibration	0.8	0.5–1	Calibrate scanner with step‐wedges or reference targets; monitor channel linearity; keep calibration logs.
	Scanning mode and orientation	0.8	0.5–1	Fix and mark film orientation; use a scanning template; always scan in the same mode (transmission/reflection) and resolution.
	Newton's rings artifacts	0.8	0.5–1	Use anti‐Newton mounts (glass backing), place protective cover, average multiple scans.
Data Processing Uncertainties	Calibration curve fitting	1.5	1–2	Use adequate calibration points spanning the dose range, choose appropriate fit (polynomial/logistic), weight points, and propagate fit uncertainty.
	Correction methodologies	0.8	0.5–1	Apply validated corrections (triple‐channel, lateral response, etc.); validate against reference measurements; report methods.
	Pixel value to dose conversion	1.5	1–2	Use 16‐bit scans when possible; avoid saturated pixels; use channel‐specific calibrations or weighted channel fusion; average ROIs.

### 6.2 Irradiation‐related uncertainties

The irradiation‐related uncertainties in Table 6 arise from the process of delivering radiation to the film. Dose delivery accuracy is estimated at 1.5%, representing the uncertainty in the radiation source's output (e.g., a linear accelerator). For well‐calibrated systems, TG‐235 (Section 3) and standards like IAEA TRS‐398^161^ indicate a 1%–2% range, with van Battum et al.^142^ confirming ∼ 1.5% for megavoltage photon beams, making this value accurate. Setup variability, also at 1.5%, accounts for errors in film positioning, phantom setup, or beam alignment during irradiation. TG‐235 suggests 1%–2%, and Palmer et al.^162^ report a 0.9% uncertainty for precise setups, indicating that 1.5% is realistic for typical clinical scenarios. Energy dependence is assigned at 2.0%, reflecting the film's varying response to different radiation energies. While minimal for megavoltage beams, kilovoltage beams or brachytherapy sources increase this uncertainty. TG‐235 estimates 1%–3%, and Sorriaux et al.^16^ report ≤1.5% for photon/proton beams, supporting the 2.0% as a balanced estimate for general use.

### 6.3 Readout system uncertainties

The readout system uncertainties in Table 6 stem from the equipment and methods used to scan the film and measure its response. Scanner variability is estimated at 1.5%, caused by variations in flatbed scanners (e.g., Epson 11000XL) due to light source instability, temperature effects, or lateral response artifacts. TG‐235 suggests 1%–2% without corrections, and León Marroquin et al. identify scanner uniformity as a key factor with similar uncertainties, validating the 1.5% estimate with proper QA.^163^ Scanner calibration contributes 0.8%, reflecting errors from inaccurate calibration of grayscale or color channels. TG‐235 estimates 0.5%–1%, and Aland et al. note that regular calibration keeps this low, supporting the 0.8% value.^164^ Scanning mode and orientation, also at 0.8%, accounts for differences between transmission/reflection modes or inconsistent film orientation. TG‐235 suggests 0.5%–1%, and León Marroquin et al. confirm that standardizing orientation limits this to ∼ 1%, making 0.8% appropriate.^163^ Newton's Rings artifacts contribute 0.8%, occurring in films like EBT2 due to interference patterns during scanning. TG‐235 estimates 0.5%–1% if uncorrected, and modern films like EBT3 reduce this issue, but the 0.8% value is fair for older models, as noted by Devic et al.^10^

### 6.4 Data processing uncertainties

The data processing uncertainties in Table 6 arise from converting scanned data into dose measurements. Calibration curve fitting is estimated at 1.5%, reflecting the uncertainty introduced when fitting a non‐linear dose‐response curve for RCFs. TG‐235 suggests 1%–2% with robust methods, and Sorriaux et al. report ≤1.5% for photon/proton beams, confirming the 1.5% estimate.^16^ Correction methodologies contribute 0.8%, as techniques like triple‐channel dosimetry or LRA correction introduce minor uncertainties if not optimized. TG‐235 estimates 0.5%–1%, and León Marroquin et al. note that advanced corrections keep this low, supporting the 0.8% value.^58^ Pixel value to dose conversion is assigned 1.5%, reflecting errors in converting scanner pixel values to dose, particularly across RGB channels. TG‐235 suggests 1%–2%, and León Marroquin et al. report channel‐specific uncertainties (e.g., 3.2%–5.2%), with pixel conversion as a key factor, making 1.5% a balanced estimate.^163^

### 6.5 Combined uncertainty and validation

To further validate Table 6, the individual uncertainties can be combined using the root‐sum‐square method: = √(2.9^2^ + 1.5^2^ + 2.0^2^ + 0.8^2^ + 1.5^2^ + 1.5^2^ + 2.0^2^ + 1.5^2^ + 0.8^2^ + 0.8^2^ + 0.8^2^ + 1.5^2^ + 0.8^2^ + 1.5^2^) ≈ 5.3%. This combined uncertainty aligns with literature findings, such as van Battum et al. reporting 1.8% (single film) under highly controlled conditions,^142^ León Marroquin et al. reporting 3.2%–5.2% for EBT3,^163^ and León Marroquin et al. reporting ∼ 3.1% for EBT‐XD.^58^ The 5.3% total is slightly conservative, appropriately covering typical clinical conditions with modern films. The table's reliability is reinforced by its alignment with TG‐235, corroboration by peer‐reviewed studies, and consistency with standard dosimetry practices.

The uncertainty estimates in Table 6 are accurate and well‐supported by the AAPM TG‐235 report, which provides guidance on RCF dosimetry uncertainties, and by studies such as van Battum et al.^142^, Sorriaux et al.^16^, and León Marroquin et al.^58^, ^163^, which report comparable values. The estimates reflect best practices (e.g., using EBT3/EBT‐XD films, calibrated scanners, and 24‐h post‐irradiation scanning) and are realistic for clinical settings. The combined uncertainty of ∼ 5.3% matches real‐world results, confirming the table's validity for guiding RCF dosimetry applications.

## 7 OVERVIEW OF CLINICAL APPLICATIONS OF RCFS

RCFs are indispensable tools in clinical radiation therapy due to their high spatial resolution, water‐equivalence, and versatility across a range of radiation modalities and dose levels (Table 7). RCFs are particularly valuable for patient‐specific QA and in vivo dosimetry. This section outlines the primary clinical uses of RCFs and focuses on their role in ensuring accurate and safe radiation delivery.

**TABLE 7:** Examples of clinical applications of RCFs.

Clinical Application	Specific Procedure/Context	RCF Model	Rationale for Selection/consideration
Machine QA	Mechanical/radiations congruence assessment, MLC test, Leakage test	EBT3/4	Ease‐of‐use, large detection area, no need for post‐processing.
Patient‐Specific QA and special procedures	IMRT, VMAT, SBRT, SRS (small fields, steep dose gradients)	EBT3/4, EBT‐XD	High spatial resolution and dynamic range (0.01–40 Gy). EBT‐XD is preferred for doses > 10 Gy. MD‐V2 for 40–100 Gy and HD‐V2 for > 100 Gy.
In Vivo Dosimetry	Total Skin Electron Therapy	EBT3/4	Surface dose measurements with 3%–5% uncertainty when properly calibrated.
	Brachytherapy (HDR, steep dose gradients)	EBT3/4	EBT3/4 for general use.
Electron Therapy	Depth dose and profile measurements, TSET commissioning and QA.	EBT3/4	Minimal energy dependence (< 4%) for electron beams (6–20 MeV), high spatial resolution for steep dose gradients, and accuracy within 2% in water‐equivalent phantoms. Suitable for standard therapeutic doses.
Proton Therapy	Lateral and transverse dose profiles, 2D/3D dose distributions, dose verification, small field dosimetry, and pencil beam spot scanning	EBT3/4	High spatial resolution and dynamic range. LET dependency must be evaluated by the user.
Image‐Guided Radiation Therapy	kV‐IGRT (e.g., CBCT)	XR‐QA2	Designed for low‐dose range (0.1–20 cGy), ideal for kV imaging surface doses.
	MV‐IGRT (portal imaging)	EBT3/4	Suitable for MV imaging doses with 2%–3% uncertainty.
Brachytherapy	Low dose‐rate (LDR) Sources	EBT3/4	Thin profile and water‐equivalence minimize perturbation around sources.
	HDR Sources	EBT3/4	Effective for verifying dwell times and positions with < 2% discrepancy from planned doses.

### 7.1 Patient‐specific quality assurance

Patient‐specific quality assurance (PSQA) is essential in radiation therapy to ensure that individual treatment plans are delivered accurately, particularly for complex techniques such as IMRT, VMAT, SRS, and SBRT.^165^ RCFs can be utilized for PSQA due to their ability to measure 2D dose distributions with high spatial resolution, although ionization chamber‐ and diode‐based array detectors are more commonly used for PSQA. This section reviews the use of RCFs in PSQA, focusing on their role in verifying dosimetric accuracy and spatial precision of treatment delivery.

RCFs are employed to confirm the dosimetric accuracy of treatment planning system calculations by measuring the delivered dose in phantoms and comparing it to the planned dose. Their high resolution, capable of detecting submillimeter variations, makes them ideal for evaluating small fields and steep dose gradients, which are common in SRS and SBRT fields.^166^, ^167^ For IMRT and VMAT delivery techniques, RCFs verify the accuracy of modulated dose distributions, ensuring that the delivered dose aligns with the planned dose within acceptable tolerances, typically assessed using gamma analysis.^168^, ^169^ RCFs have been extensively utilized in PSQA across a range of advanced radiation therapy techniques, playing a critical role in ensuring the accuracy and safety of complex treatments. In IMRT and VMAT, RCFs validate the precision of modulated beams by detecting errors in multileaf collimator positioning or beam modulation, which are essential for achieving conformal dose delivery.^51^, ^168^, ^169^ Studies have employed RCFs confirming dose distributions in these modalities, including with flattening filter‐free beams.^170^, ^171^ For SRS and SBRT, RCFs provide dose measurement in small fields (e.g., 4–10 mm) and verify the spatial accuracy of high‐dose delivery, ensuring that steep dose gradients effectively spare surrounding tissues, as demonstrated through end‐to‐end tests for linac‐based SRS commissioning and CyberKnife systems.^172^, ^173^ In non‐isocentric treatments and those involving moving targets such as with TomoTherapy, RCFs support PSQA by verifying spatial delivery and tracking accuracy, and they are also instrumental in measuring doses in dynamic phantoms for moving structures, like the prostate or lung tumors.^174^, ^175^ Additionally, in Gamma Knife SRS, RCFs are leveraged to confirm the geometric and dosimetric accuracy of treatment plans, including the unit center point (UCP) and the spatial precision required for treatments such as trigeminal neuralgia, where their submillimeter resolution is particularly advantageous.^173^, ^176^

### 7.2 In vivo dosimetry

In vivo dosimetry with RCFs provides dose verification during treatment.^177^ RCFs have been used for surface dose measurements in total skin electron therapy (TSET),^178^ intraoperative electron radiotherapy,^179^ and intraoperative brachytherapy.^180^ In TSET, RCFs assess skin doses across large areas, with uncertainties of ∼3%–5% when accounting for film orientation and calibration.^181^ In brachytherapy, particularly with high dose‐rate (HDR) sources, RCFs verify dose distributions around applicators or interstitial needles, leveraging their submillimeter resolution for steep dose gradients.^180^ Recently, a clinical study comparing EBT3 and EBT‐XD films in high‐energy electron beam radiation therapy for keloid patients found that EBT‐XD provided better agreement with the prescribed dose, with higher accuracy and lower mean dose differences suggesting that EBT‐XD may be a more suitable choice for hypofractionated surface dose verification in clinical settings due to its extended dynamic range.^182^

### 7.3 Image‐guided radiation therapy

Image‐guided radiation therapy (IGRT) enhances radiation therapy quality by enabling accurate patient positioning and real‐time monitoring of tumor changes during treatment. Technologies such as cone‐beam computed tomography (CBCT) and spiral CT are integral to IGRT, ensuring precise dose delivery by adapting to variations in tumor position, size, and shape.^183^ RCFs have been utilized in both kV and MV IGRT procedures for dosimetry and QA, offering high spatial resolution and practical application. Their applications in dose verification, geometric precision, and system performance evaluation enhance the reliability of IGRT, ultimately improving treatment accuracy and patient outcomes. In kV IGRT, CBCT, and spiral CT technologies are employed for patient setup and anatomical verification. Accurate imaging is crucial for these low‐energy imaging techniques and RCF dosimetry provide a robust method for achieving this.^184^ The XR models of RCF, which include high atomic number (*Z*) components in the sensitive layer, enhance film response at low energies via photoelectric interactions, making them well‐suited for kV imaging.^185^ Established protocols for CBCT dosimetry using RCF are documented in the literature, with a detailed review of reference RCF dosimetry for kV IGRT procedures provided by Devic et al.^10^ RCF LD‐V1 proved feasible and effective for measuring dose profiles in wide detector CT scanners, offering a more comprehensive assessment than the console‐displayed CTDI_vol_ alone.^186^ In kV IGRT procedures, where CBCT often employs wide beams, this approach enhances dosimetry accuracy. The high spatial resolution of RCF makes it particularly suitable for capturing detailed dose distributions, which is critical for verifying imaging doses and ensuring patient safety during treatment.

While kV CBCT is the most common imaging modality, some IGRT systems utilize MV CT (MVCT) with photon beams from linac‐based x‐rays.^183^ Reports suggest MVCT dose, typically ranging from 0.01 to 0.03 Gy per scan, using RCF or diode arrays for comparison to baseline values.^187^, ^188^ Additionally, RCF supports optimization of the volume‐of‐interest to minimize CT dose. For example, EBT2 film was used to measure dose from an MVCT source with a carbon target and 2.35 MV accelerating voltage.^189^ The mechanical accuracy of the isocenter in CBCT systems can also be assessed using the star pattern test, where RCF is particularly effective.^190^

### 7.4 Brachytherapy

Brachytherapy dosimetry demands submillimeter spatial resolution and flexible detector geometry to capture steep dose gradients near small, curved sources. RCFs fulfill these requirements with their tissue‐equivalent response, low light sensitivity, and ability to be cut and shaped for precise placement in solid or liquid phantoms.^11^ Early RCF models (e.g., MD‐55, HD‐810) required lengthy exposures, limiting measurements to within a few millimeters of radioactive sources such as ^106^Ru/^106^Rh eye plaques, ^192^Ir, and ^125^I.^191^, ^192^, ^193^ Since the introduction of the EBT series in 2004, irradiation times have been drastically reduced, enabling accurate dose mapping up to 5 cm from seeds and plaques in water‐equivalent phantoms.^194^ Unlaminated EBT3 films have even been curved to conform to ocular plaque surfaces for surface‐dose measurements in specialized eye phantoms.^195^

For HDR sources (e.g., ^192^Ir), RCFs such as RTQA2 and EBT2/3 have been used for dose distribution measurements, TG‐43 parameter derivation, and QA of dwell positions and times in remote afterloaders and applicators.^196^, ^197^, ^198^ In intravascular brachytherapy, it was reported that RCFs could resolve gradients (> 50% per mm) and validate Monte Carlo simulations and heterogeneity effects.^199^, ^200^ RCFs such as HD‐810, MD‐55, and HS were used for studying heterogeneity effects such as calcium, stents, gold markers, air pockets, and guidewires in intravascular brachytherapy source dosimetry.^11^, ^201^, ^202^

Recently, Palmer et al. compared the new EBT4 film to EBT3 across VMAT prostate, stereotactic ablative radiotherapy (SABR) lung, and HDR brachytherapy plans.^203^ EBT4 demonstrated a ∼ 46% higher signal‐to‐noise ratio at 500 cGy and similar energy response, scan‐orientation, and darkening behaviors as EBT3. Clinically, EBT4 achieved equal or better gamma pass rates (e.g., VMAT 3%/3 mm: 100% vs. 97.9%; SABR 2%/2 mm: 99.6% vs. 97.9%; HDR 3%/2 mm: 97.7% vs. 95.0%). These results establish EBT4's superior noise profile and dosimetric accuracy for radiotherapy QA.

### 7.5 Electron therapy

RCFs have been widely utilized in electron beam dosimetry. RCF calibration for electron beams demonstrates robust performance across various conditions. For energies between 6 and 20 MeV, energy dependence is minimal, less than 4% in phantom.^84^ Dose rate dependency has negligible impact, and depth dependence is insignificant up to the depth of 50% dose.^102^, ^204^ Comparative studies show that PDD measurements with EBT2 films in water align within 2% of ionization chamber results.^205^ Similarly, EBT3 films exhibit calibration uncertainties of approximately 2% for electron beams and 1.5% for photon beams.^16^ These findings confirm RCF's reliability when validated against standard tools like ionization chambers, diodes, and Monte Carlo simulations.

Accurate RCF dosimetry in electron beams demands meticulous setup. At shallow depths (0–5 mm in water), dose deviations may occur due to air‐water perturbations or secondary electrons from the collimator head or applicator.^206^ In polystyrene phantoms, EBT films placed parallel to the beam axis can show surface dosimetric uncertainties from small air pockets, though these issues are manageable with careful handling.^207^ Additionally, a dose pulse dependence is noted for EBT films at 3–9 MeV, with pulses ranging from 30 to 70 mGy/pulse; however, this remains within 5% of the absolute dose in water for clinically relevant ranges.^208^ RCF excels in diverse electron therapy tasks including TSET: ensures dose uniformity and delivery precision^178^, ^209^, ^210^; Intraoperative electron RT: facilitates accurate dosimetry during procedures^211^; Small field dosimetry: measures doses for fields as small as 1 cm with high precision^212^; Specialized studies: evaluates dose perturbations from eye shields and surgical clips.^213^, ^214^

### 7.6 Proton therapy

The dose‐response behavior of RCF in proton beams has been well‐documented.^52^ The stopping power ratios between water and RCF remain constant with depth in water, making RCF suitable for measuring depth dose distributions and lateral dose profiles, provided appropriate corrections for LET are applied.^215^ RCFs have been employed in proton therapy for various tasks including: Mechanical alignment checks: ensuring precise beam delivery^216^; Lateral and transverse dose profiles: measuring beam spread and uniformity^217^; Dose profiles with multi‐leaf collimators: assessing shaped dose distributions^218^; Longitudinal dose distributions: evaluating dose along the beam path^113^; Spatial LET distributions, output factors, and PDD: characterizing beam properties^18^, ^52^, ^219^; 2D/3D dose distributions: Mapping complex dose patterns^217^; Dose perturbations and artifacts from implants: studying effects of fiducials, titanium, spinal implants, or tantalum markers^220^, ^221^, ^222^, ^223^; Dose verification: confirming delivered doses^224^; Patient‐specific 2D/3D dose verification: tailoring treatment validation^225^, ^226^; Fabrication of wax bolus and compensators: enhancing beam modulation with 3D printing.^227^, ^228^ RCFs also support QA through its use in anthropomorphic phantoms by the Imaging and Radiation Oncology Core (IROC), distributed to proton therapy centers for QA and treatment planning system comparisons.^229^, ^230^ It confirms consistent range with beam shaping apertures^231^ and verifies passive scattering range compensators in proton radiography.^232^ PDD measurements investigated with RCF when oriented parallel with respect to the proton beam axis.^18^ It has been found that the energy response varies with position in the Bragg peak due to continuous energy loss, with an empirical LET correction improving agreement with ion chamber data. Parallel orientation allows capturing a full 2D dataset from one film, though air columns in solid phantoms can distort entrance doses, Bragg peaks, and SOBPs. To mitigate this, angling the beam by 2–3 degrees or placing the film in water is recommended. Dosimetry becomes more complex in small proton fields and pencil beam spot scanning, where the Bragg peak's shape changes with field size.^18^ RCFs can measure 2D dose distributions and the full‐width at half‐maximum (FWHM) of pencil beam spots during commissioning,^233^ and capture the low‐dose envelope of spot profiles.^234^

Recent studies on EBT‐XD and EBT3 films under high‐dose proton beams (up to 45 Gy) demonstrate that EBT‐XD offers superior lateral response and is better suited for ultra‐hypofractionated treatments (> 20 Gy), such as FLASH and Arc proton therapy, while EBT3 is adequate for standard doses (< 20 cGy).^235^ Emerging technologies enhance RCF's role in proton therapy. A novel 3D radiochromic dosimeter, combining hydrogel photonic crystals with film stacking or 3D printing, offers a dose sensitivity of 10 nm Gy^−1^ and spatial resolution < 50 µm.^236^ This system detects complex 3D topographical dose distributions through radiation‐induced polymer cross‐linking, with tunable sensitivity and rapid readout, making it ideal for clinical dose verification. In conclusion, RCF's versatility and precision make it a cornerstone of proton therapy, supporting a wide range of dosimetric tasks from basic beam analysis to advanced patient‐specific verifications. Like other radiation types, EBT3 and EBT4 RCF models are recommended for proton therapy dosimetry applications involving doses smaller than approximately 10 Gy; for higher doses up to ∼40 Gy, EBT‐XD is the preferred model. For a given task, the films should be calibrated in beam‐matched conditions; users should apply LET/beam‐quality corrections near the Bragg peaks, correct for scanner lateral response, and perform beam‐matched validation in UHDRs for FLASH RT dosimetry before using films for quantitative dosimetry.

## 8 SUMMARY

Radiochromic films are convenient dosimeters for radiation therapy use, distinguished by their unparalleled spatial resolution and versatility in capturing two‐dimensional dose distributions. RCFs replaced traditional radiographic films due to their self‐developing nature, near‐water‐equivalence, and broader dynamic range. These attributes make RCFs indispensable for applications ranging from patient‐specific QA in complex modalities to in vivo dosimetry, proton therapy, and emerging FLASH RT. The high resolution of RCFs is particularly critical for small field dosimetry, where precise measurement of steep dose gradients ensures treatment accuracy and patient safety. The evolution from radiographic to RCFs reflects significant technological advancements, with modern RCFs offering improved uniformity, energy independence, and sensitivity across diverse radiation types, including photons, electrons, and protons. Robust calibration protocols enable dosimetric uncertainties of ∼ 2%–5%. However, achieving such accuracy demands meticulous attention to film handling, storage, orientation, and readout systems, as well as rigorous uncertainty management.

Looking forward, ongoing innovations in RCF technology promise to further expand their utility in next‐generation radiation therapy techniques. As radiation therapy continues to evolve with advancements like MRI‐guided and FLASH radiation therapies, RCFs will remain vital for validating complex dose distributions and ensuring dosimetric fidelity in clinical and preclinical settings. By combining established best practices with emerging developments, film dosimetry will continue to play a pivotal role in advancing the precision, safety, and efficacy of radiation therapy.

## CONFLICT OF INTEREST STATEMENT

The authors declare no conflict of interest.
